# Targeting Wnt/β-Catenin Signaling by TET1/FOXO4 Inhibits Metastatic Spreading and Self-Renewal of Cancer Stem Cells in Gastric Cancer

**DOI:** 10.3390/cancers14133232

**Published:** 2022-06-30

**Authors:** Jingjing Qi, Di Cui, Qi-Nian Wu, Qi Zhao, Zhan-Hong Chen, Lianjie Li, Walter Birchmeier, Yong Yu, Ran Tao

**Affiliations:** 1 General Surgery, Cancer Center, Department of Hepatobiliary & Pancreatic Surgery and Minimally Invasive Surgery, Zhejiang Provincial People’s Hospital (Affiliated People’s Hospital, Hangzhou Medical College), Hangzhou 310014, China; jingjing.qi@jku.at (J.Q.); cuidi@hmc.edu.cn (D.C.); lilianjie@bestandcro.com (L.L.); 2 Tumor Epigenetics Laboratory, Johannes Kepler University Linz, Altenbergerstraße 69, 4040 Linz, Austria; 3 Cancer Center, Department of Gastroenterology, Zhejiang Provincial People’s Hospital (Affiliated People’s Hospital, Hangzhou Medical College), Hangzhou 310014, China; 4 State Key Laboratory of Oncology in South China, Collaborative Innovation Center for Cancer Medicine, Sun Yat-sen University Cancer Center, Guangzhou 510060, China; wuqn@sysucc.org.cn (Q.-N.W.); zhaoqi@sysucc.org.cn (Q.Z.); 5 Department of Oncology, The Third Affiliated Hospital of Sun Yat-sen University, 600 Tianhe Road, Guangzhou 510630, China; chzhanh3@mail.sysu.edu.cn; 6 Max-Delbrück-Center for Molecular Medicine in the Helmholtz Association, 13125 Berlin, Germany; wbirch@mdc-berlin.de

**Keywords:** gastric cancer, TET1, FOXO4, Wnt signaling, cancer stem cell, metastasis

## Abstract

**Simple Summary:**

Metastasis is the main cause of death for patients suffering gastric cancer. Epithelial-mesenchymal transition (EMT) and cancer stem cells (CSC) are critical attributes of metastasis, both of which are regulated tightly by DNA methylation and Wnt/β-catenin signaling. In this study, we unveiled a novel TET1-FOXO4-β-catenin signaling cascade, in which TET1 inhibits β-catenin activity and its nuclear translocation through transactivating FOXO4 expression. TET1 expression can significantly inhibit EMT and stemness properties of gastric cancer cells, while knocking-down endogenous TET1 induces metastasis and enhances self-renewal of CSCs by activating canonical Wnt signaling, which could be fully rescued by modulating FOXO4 expression. Our data also showed that low expression of TET1 or FOXO4 predicts poor survival of gastric cancer patients, suggesting reactivation of TET1 or FOXO4 might be a novel therapeutic approach to prevent gastric cancer metastasis.

**Abstract:**

Metastasis is the main cause of death for patients suffering gastric cancer. Epithelial-mesenchymal transition (EMT) and cancer stem cells (CSC) are critical attributes of metastasis, both of which are regulated tightly by DNA methylation and Wnt/β-catenin signaling. Here, we studied the functions of DNA dioxygenase TET1 in regulating Wnt signaling and in gastric cancer metastasis. Knocking-down and overexpressing TET1 in gastric cancer cells promoted and inhibited metastatic spreading to the liver in immune-deficient mice, respectively. TET1 showed inhibitory effects on metastasis-related features -EMT and CSC, which were reversed by interfering with Wnt/β-catenin signaling. RNA-sequencing identified FOXO4 as a direct transactivating target of TET1. FOXO4 directly interacted with β-catenin and recruited it in the cytoplasm, so as to inhibit β-catenin-mediated transcription of Wnt target genes, including CSC marker EpCAM. Moreover, modulation of FOXO4 could reverse the effects of TET1 manipulation on EMT and self-renewal of CSCs. The analysis with clinical samples confirmed the value of FOXO4 as an independent prognostic predictor of patients’ overall survival. Taken together, regulation of Wnt signaling by TET1/FOXO4 is essential for metastasis-associated cellular properties, and targeting TET1/FOXO4/β-catenin pathway may serve as promising therapeutics in the prevention and treatment of gastric cancer metastasis.

## 1. Introduction

Gastric cancer is a prevalent disease, with over 769,000 deaths reported annually [1]. Although surgery and chemotherapy have significantly improved patients’ survival, therapeutic effects are still not optimal, due to relapse and metastasis [2]. During cancer metastasis, epithelial-mesenchymal transition (EMT) is a critical step that allows polarized epithelial tumor cells to become mesenchymal-like, enhances cell migration and invasion, and generates stem cell-like properties [3,4]. Cancer stem cells (CSCs) are able to self-renew and differentiate into all parts of a tumor [4], which are considered to be the “seeds” of metastases. Therefore, the cancer research community engages in finding effective ways to suppress EMT and self-renewal of CSCs, both of which are controlled by complicated and delicate crosstalks between extracellular molecules, such as growth factors and cytokines, and intracellular signaling systems, for which epigenetic modification is critical [5,6].

DNA methylation has been shown to play an important role in promoting EMT and maintaining the self-renewal of CSCs [7,8]; however, little is known about whether DNA demethylation can maintain epithelial properties or inhibit CSCs’ expansion. DNA methylation of promoters regulates gene transcription epigenetically, DNA methyltransferases (DNMTs) promote cytosine methylation in the promoters, thus inhibit gene transcription; DNA methylcytosine dioxygenases TET family proteins convert DNA methylation at the 5′ position of the cytosine base (5mC) primarily to 5-hydroxymethylcytosine (5hmC) and subsequently to 5-formylcytosine or 5-carboxylcytosine [9,10]. Low 5hmC levels exist in a variety of solid tumors and cancer cell lines [11,12,13,14], implying a tumor-suppressive function of DNA hypomethylation and TET1. Genetic mouse models have shown that TET family proteins execute suppressive functions in cancer initiation and progression [15,16]; however, whether TET1 can regulate metastasis and associated cellular features, such as EMT and self-renewal of CSCs, is still unknown.

Aberrant canonical Wnt signaling is fundamental in gastric cancer development [17]. Activation of Wnt/β-catenin signaling is also closely related to EMT and expansion of CSCs [18]. β-Catenin is the key molecule of Wnt signaling, of which localization and activity is tightly controlled in normal cells by cell adhesion molecule E-cadherin, scaffold protein APC and inhibitory kinases GSK3β and CK1α [19,20]. However, APC inactive or hyperactive β-catenin mutants may drive aberrant activation of β-catenin and excessive transcription of Wnt downstream targets, including c-Myc, Cyclin D1, EpCAM, Snail, and Twist, which consequently cause physiological and pathological disorders, including cancer [20,21]. Therefore, the inhibition of β-catenin, the crucial intracellular messenger of Wnt signaling transduction, is attracting constant interest in clinical cancer research [22].

This study aims to investigate the role of TET1 in metastasis and associated cellular features, EMT and CSCs, alongside the objectives of identifying novel prognostic markers of gastric cancer. In the present study, we found that knocking-down TET1 promoted experimental metastasis in mice, while TET1 overexpression inhibited cancer cell seeding to livers. Furthermore, knocking-down TET1 induced EMT and enhanced self-renewal of CSCs in tissue culture. In contrast, TET1 overexpression preserved epithelial features of gastric cancer cells and decreased expansion and frequencies of CSCs. We also identified FOXO4, a negative regulator of Wnt/β-catenin signaling, as a direct demethylating target of TET1. In addition, modulation of FOXO4 expression rescues the inhibitory effects of TET1 on gastric cancer cells. Thus, our results unveiled a novel epigenetic regulation of Wnt/β-catenin signaling by TET1 as a DNA demethylase and suggested therapeutic application of Wnt inhibitors to target EMT and CSCs as a promising strategy to suppress metastasis, as well as examining the expression of TET1 and FOXO4 during the diagnosis of gastric cancer to predict the cancer progression and warrant more aggressive treatments in certain cases.

## 2. Materials and Methods

### 2.1. In Vivo Experimental Metastasis Assay

A total of 25 6–8 weeks old male NOD/SCID mice were randomly allocated into the following 6 groups: MKN28, MKN28_Ctrl, MKN28_shTET1, MKN45, MKN45_Ctrl, MKN45 _TET1 (see Figure 2 and Figure S3). Minimal sample size was determined based on experience and ethical regulations. Next, 1 × 10^6^ of MKN28, MKN45 or shTET1/TET1-transduced cell lines were suspended in 50 µL PBS and injected into the spleens as described [23]. After three (for MKN28_shTET1, MKN45 and MKN45_Ctrl cells, which had higher proliferation rates) or four weeks (for MKN28, MKN28_shCtrl, and MKN45_TET1 cells, which showed slower proliferation), another investigator blinded to the grouping examined the mice. Spleens and livers were snap-frozen in liquid N2 to isolate RNA or fixed in 4% formaldehyde to prepare paraffin-embedded blocks. Pictures of freshly isolated organs were recorded with a Samsung digital camera.

### 2.2. Cell Culture, Plasmids, and Retroviral Gene Transfer

All cell lines were purchased from ATCC, and yearly authenticated by STR profiling in the Department of Forensic Medicine of Sun Yat-sen University. Mycoplasma contamination was examined by PCR every month. Cells were cultivated in DMEM supplemented with 10% FBS, 100 U/mL penicillin and 100 µg/mL streptomycin. pSuper-retro system was used to stably knockdown TET1 and FOXO4 gene expression. shRNA sequences are ACACAACTTGCTTCGATAATT (TET1) and ACCGTGAAGAAGCCGATATGT (FOXO4). Retroviral gene transfer and antibiotic selection of cell lines were previously described [24].

### 2.3. Cell Proliferation Assay

For this step, 2 × 10^4^ or 1 × 10^5^ stable cells were plated in 24-well or 12-well plates, respectively, and counted at the indicated time points. Cell numbers were measured using Guava ViaCount assay on Guava easyCyte Flow Cytometer system (Millipore, Darmstadt, Germany) and analyzed with the Guava CytoSoft software package.

### 2.4. In Vitro Tumorsphere Formation Assay

Gastric cancer cells were plated in poly(2-hydroxyethyl methacrylate)-coated (Sigma, Darmstadt, Germany, P3932) plates at a density of 1000 cells/mL. Cells were grown in serum-free DMEM/F12 medium (Gibco), supplemented with B27 (Gibco, Grand Island, NY, USA, 17504044), 20 ng/mL FGF (R&D, McKinley Place N.E., Minneapolis, MN, USA, 233-FB), 20 ng/mL EGF (R&D, 236-EG) and 4 μg/mL heparin (Sigma, 9041-08-1) for 1–2 weeks. The number of tumorspheres was counted and the images were captured using a phase contrast microscope. Results are presented as percentages of numbers of spheres generated per 100 cells seeded and spheres with diameter >100 µm accordingly.

### 2.5. Transwell Cell Migration Assay

Single cell migration was evaluated by using 8 μm polycarbonate nucleopore filter-containing Boyden chambers (Millipore, Darmstadt, Germany, PIEP15R48/PIXP01250). Next, 10^4^ cells suspended in 100 μL of normal culture medium were plated in the upper chamber for 6–8 h to attach. Medium without FBS was changed in the upper chamber, while complete media (containing 10% FBS) was added to the lower chamber. After 24 h of incubation at 37 °C, migrated/invaded cells were fixed by 4% formaldehyde and stained with 0.005% crystal violet. Cells were counted in 4 separate fields per filter.

### 2.6. Immunoblotting, Immunofluorescence and Immunoprecipitation

Immunoblotting (IB) and immunofluorescence staining (IF) were performed as previously described [17]. The immunoprecipitation (IP) experiment was performed using 10^7^ cells for each IP reactioin with Dynabeads™ Protein G Immunoprecipitation kit (Invitrogen, Vilnius, Lithuania, Cat. No 10007D) according to the manufacture’s instruction. For detailed information regarding antibodies, please refer to Table S2.

### 2.7. Chromatin Immunoprecipitation (ChIP)

ChIP-qPCR assay was performed as previously described [23]. Briefly, ChIP assays were performed using the iDeal ChIP-seq Kit (Diagenode, Denville, NJ, USA, C01010170) and antibodies against TET1 or IgG as the negative control. Lysate of 5 × 10^6^ cells was used for each ChIP reaction. Precipitated DNA samples were analyzed by quantitative PCR with primer pairs specific to the promoters of FOXO4 and PTEN genes. For detailed PCR primer sequences, please refer to Table S3.

### 2.8. Dot Blot

Genomic DNA was extracted from cells using TIANamp Genomic DNA Kit (Tiangen Biotech, Beijing, China, DP304) and denatured by incubation at 99 °C for 5 min. DNA was then spotted onto Amersham Hybond-N+ membrane (GE Healthcare), air-dried and UV-crosslinked. The membrane was blocked and incubated in primary 5hmC or 5mC antibody overnight. After secondary antibody incubation, the membrane was proceeded with ECL exposure and imaging. Dot intensity was quantified by ImageJ software.

### 2.9. Bioinformatics Analysis and Code Availability

Raw RNA sequencing reads were filtered out with the following criterions: (1) contain more than 10% Ns or low-quality bases; (2) contain adaptors or sequences from other species. Reads quality were checked using Fastqc (https://www.bioinformatics.babraham.ac.uk/projects/fastqc/) (accessed on 26 June 2022), and the filtered reads were considered as clean reads for further analysis. Differential expression analysis was performed using DESeq2 [25] under R environment.

### 2.10. Statistics

All experiments were conducted in biological triplicates unless otherwise stated. Statistical analyses were performed using Graphpad Prism 5.0 package. A significance level of *p* < 0.05 was used throughout the study. Differences between two experimental groups were analyzed using the unpaired two-tailed Student’s *t*-test; growth curves were calculated using the two-way ANOVA.

## 3. Results

### 3.1. The DNA Methylcytosine Dioxygenase TET1 Is Low in Metastatic Gastric Tumors and Predicts Poor Survival of Patients

**Figure 1:**
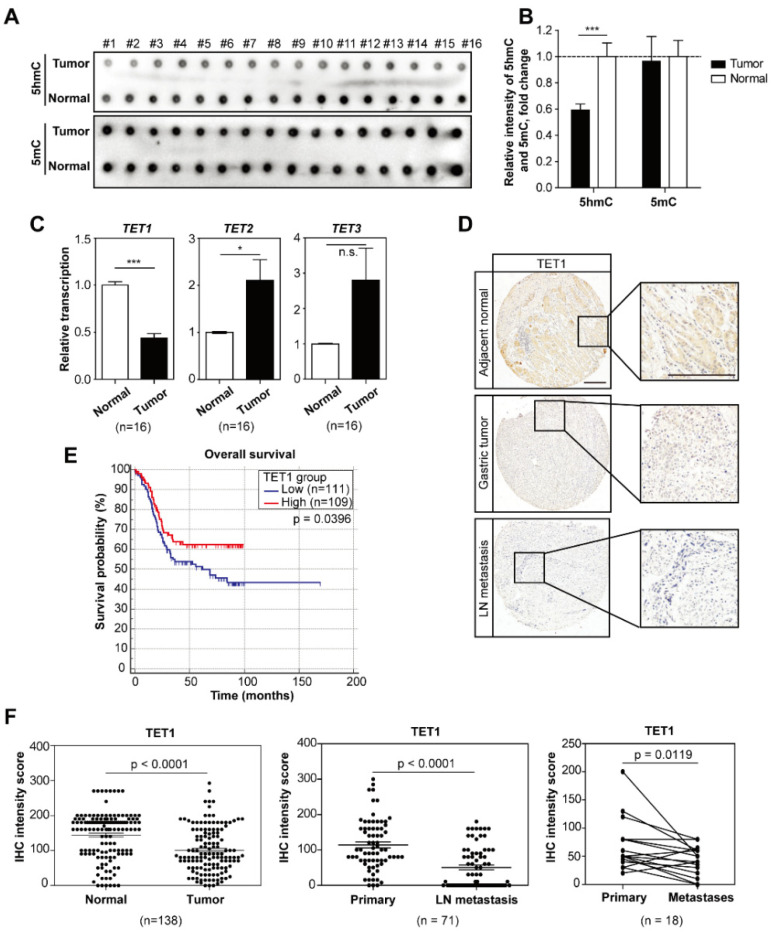
The DNA methylcytosine dioxygenase TET1 is low in metastatic gastric tumors and predicts poor survival of patients. (**A**) Dot blot analysis of global 5hmC and 5mC levels in paired tumor biopsies and adjacent normal tissues from patients with metastatic gastric cancer. (**B**) Quantification of relative 5hmC and 5mC levels in (**A**). (**C**) qRT-PCR analysis of TET1, TET2 and TET3 transcripts comparing paired tumors vs. normal tissue samples as in (**A**). (**D**) Representative pictures of TET1 IHC staining in normal tissue, primary tumors and lymph node metastases of gastric cancer patients. Scale bars represent 100 µm. (**E**) Kaplan–Meier analysis showing overall survival of gastric cancer patients with high vs. low TET1 expression. Median value of TET1 IHC intensities is used as cut-off to separate low and high groups. (**F**) IHC staining intensities comparing TET1 expression in normal tissue samples vs. paired primary tumors (**left**), primary tumors vs. paired lymph node metastases (**middle**), and primary tumors vs. paired distant metastases (**right**). Error bars indicate mean ± SD. ***, *p* < 0.001. *, *p* < 0.05. n.s., not significant.

To evaluate whether DNA methylation may affect cancer metastasis, we performed an endoscopy on 16 patients with metastatic gastric cancer and collected primary tumors and adjacent normal tissues. By dot blot analysis, we observed that 5-hydroxylmethylcytosine (5hmC) was greatly reduced in tumor samples (Figure 1A, quantified in Figure 1B). We then examined in the same sixteen pairs of samples the expression of TET1 and its two family members TET2 and TET3, which catalyze the conversion from 5mC to 5hmC. TET1 expression, but not TET2 or TET3, was lower in tumors compared to normal tissues, implying the causal correlation between TET1 and 5hmC (Figure 1C). Analysis of the TCGA dataset showed a similar trend in stomach adenocarcinomas, although it was not significant (Figure S1A).

To further confirm this, we performed immunohistochemistry (IHC) in tissue arrays of hundreds of patient samples, including samples from cancer metastases in lymph nodes and distant organs. Analyses using paired samples indeed showed low levels of TET1 expression in tumor samples, and notably, further strong reductions in lymph nodes and distant organ metastases (Figure 1D,F and Figure S1B). The Kaplan–Meier analysis confirmed that low levels of TET1 were significantly associated with poor overall survival of patients (Figure 1E). Taken together, these data suggest that TET1 is negatively associated with gastric cancer progression and metastasis.

### 3.2. Knocking-Down TET1 Promotes Experimental Metastasis, While TET1 Overexpression Inhibits Metastasis in the Liver

**Figure 2:**
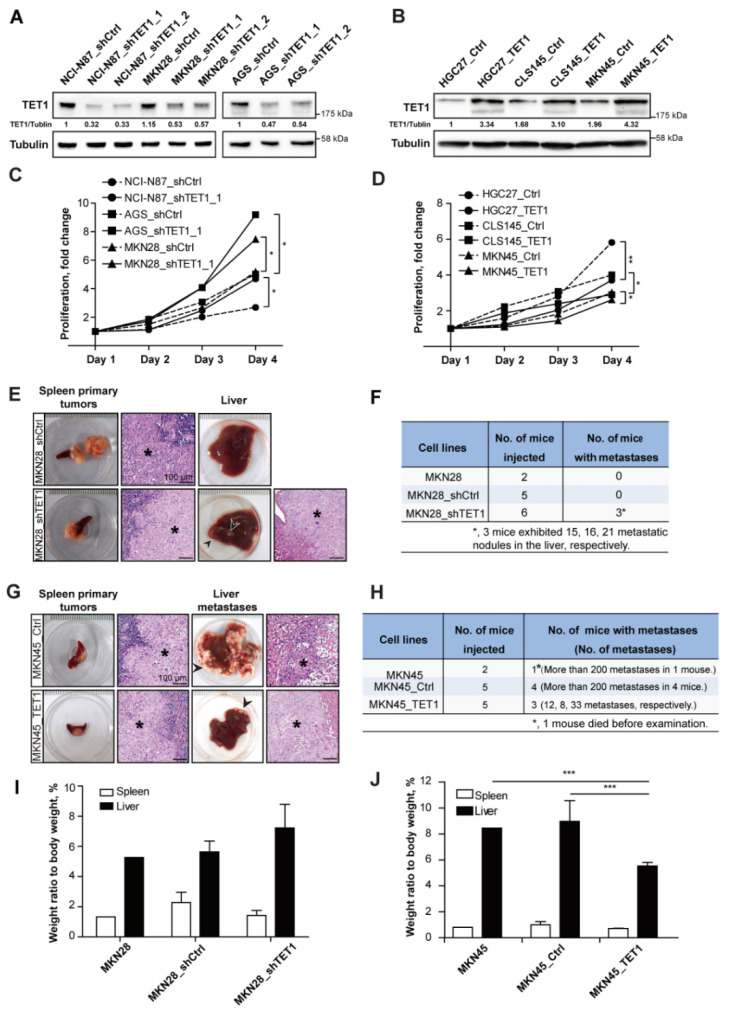
Knocking-down TET1 promotes experimental metastasis, while TET1 overexpression inhibits metastasis in the liver. (**A**) Immunoblot analysis showing TET1 expression in NCI-N87, MKN28, and AGS cells transduced with shTET1 or scrambled shRNA as control (shCtrl). α-Tubulin was used as loading control. (**B**) Immunoblot analysis showing TET1 expression in HGC27, CLS145, and MKN45 cells transduced with ectopic TET1-expressing or empty vector as control (Ctrl). α-Tubulin was used as loading control. (**C**,**D**) Relative cell proliferation rates of stable cell lines. (**E**,**G**) Representative pictures of tumors and H&E staining showing primary tumors in the spleen and metastatic tumors in the liver of mice injected with MKN28 stable cell lines (**E**) or with MKN45 stable cell lines (**G**). Asterisk represents tumor tissue. Scale bars represent 100 µm. (**F**,**H**) Case numbers of mice with metastases injected with MKN28 stable cell lines (**F**) or with MKN45 stable cell lines (**H**). (**I**,**J**) Relative weight of spleens (spleen/body weight) and livers (liver/body weight) in (**E**) or (**G**). Error bars indicate mean ± SD. ***, *p* < 0.001. **, *p* < 0.01. *, *p* < 0.05.

To understand how TET1 may regulate gastric cancer progression and metastasis, we selected and modified a series of human gastric cancer cell lines. The endogenous levels of TET1 were higher in AGS, MKN28 and NCI-N87 cells than in HGC27, MKN45 and CLS145 cells (Figure S2A,B). The expression levels of TET1 coincided with the levels of endogenous 5hmC in these cell lines (Figure S2C, quantified in Figure S2D). Knocking-down TET1 in TET1 high-expressing cells significantly reduced 5hmC level (Figure 2A and Figure S2E,G,H), while TET1-overexpressing in TET1 low-expressing cells enhanced 5hmC levels (Figure 2B and Figure S2F,I,J). In addition, shTET1 stable cell lines showed enhanced proliferation rates (Figure 2C), while TET1-overexpressing cell lines slightly reduced proliferation (Figure 2D).

We employed spleen injections to measure experimental liver metastasis of the stable cell lines [23,26]. After three (for MKN28_shTET1, MKN45 and MKN45_Ctrl) or four weeks (for MKN28, MKN28_shCtrl and MKN45_TET1), spleens were examined for primary tumors and livers for metastases. Half of the mice injected with MKN28_shTET1 cells developed multiple macroscopic liver metastases; however, after given one more week to grow, all the mice injected with MKN28 parental or control cells (MKN28_shCtrl) were metastasis-free (Figure 2E,F). In contrast to MKN45 and MKN45_Ctrl cells, overexpressing TET1 in MKN45 cells drastically reduced the numbers of metastatic nodules in the liver (Figure 2G,H). H&E staining of tumorous livers and spleens confirmed the existence of cancer cells in the lesions (Figure 2E,G). High metastatic burden in mice injected with MKN28_shTET1 cells or MKN45_Ctrl cells led to relatively heavier livers in these mice (Figure 2I,J). Intriguingly, qRT-PCR analyses revealed that TET1 expression in MKN28_shTET1 cells was further decreased, when liver metastatic nodules were compared with spleen primary tumors, indicating a causal relationship between TET1 expression levels and metastatic capability (Figure S3A). Similarly, parental MKN45 cells slightly reduced TET1 expression in the liver metastatic cells (Figure S3B). Taken together, in the experimental metastasis model, knocking-down TET1 expression promotes metastasis of gastric cancer cells, while TET1 overexpression inhibits the formation of metastatic nodules.

### 3.3. TET1 Inhibits Self-Renewal of Cancer Stem Cells and Epithelial-Mesenchymal Transition

**Figure 3:**
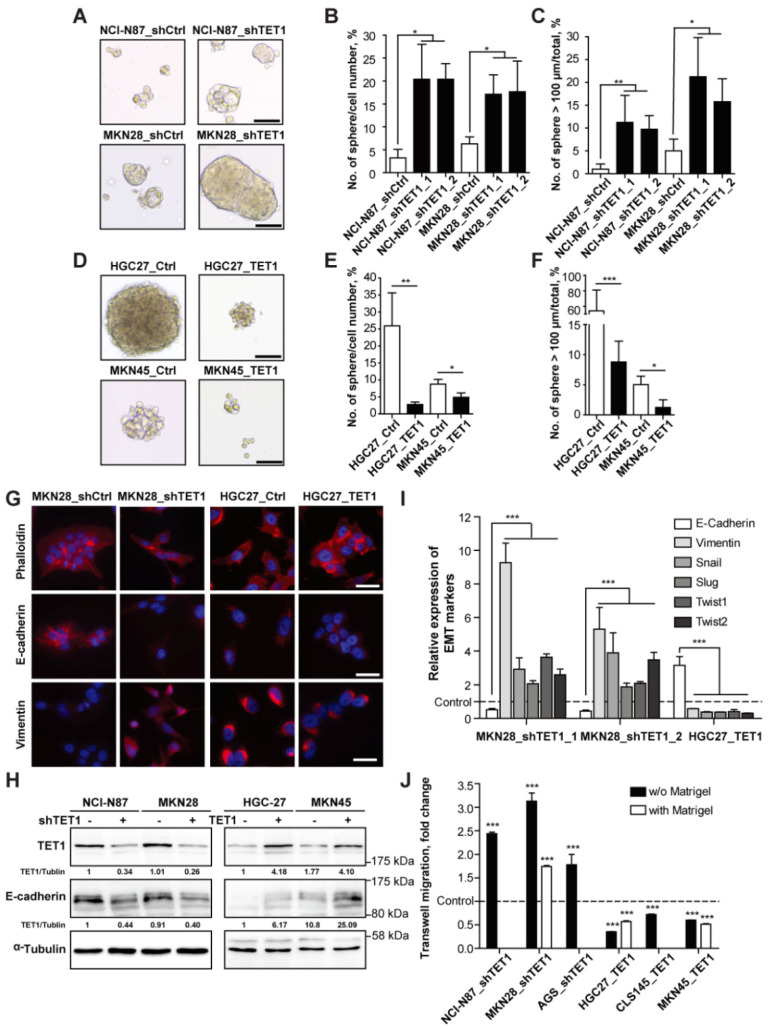
TET1 inhibits self-renewal of cancer stem cells and epithelial-mesenchymal transition. (**A**) Representative pictures of spheres generated by stable NCI-N87 and MKN28 cell lines. Scale bars represent 100 µm. (**B**) Number of spheres generated per 100 cells seeded as in (**A**). (**C**) Percentage of spheres with diameter >100 µm as in (**A**). (**D**) Representative pictures of spheres generated by stable HGC27 and MKN45 cell lines. Scale bars represent 100 µm. (**E**) Numbers of spheres generated per 100 cells seeded as in (**D**). (**F**) Percentage of spheres with diameter >100 µm as in (**D**). (**G**) Immunofluorescent staining of β-actin (by phalloidin), E-cadherin, and vimentin in MKN28 and HGC27 stable cell lines. Scale bars represent 100 µm. (**H**) Immunoblot analysis showing TET1 and E-cadherin expression in stable gastric cancer cell lines. α-Tubulin was used as loading control. (**I**) qRT-PCR analysis of the EMT-related transcripts in stable cell lines transduced with shTET1 or TET1. Respective control cells were used as control. (**J**) Quantification of transwell migration assays with or without Matrigel coating of indicated stable cell lines compared to respective control cells. Error bars indicate mean ± SD. ***, *p* < 0.001. **, *p* < 0.01. *, *p* < 0.05.

In order to characterize the impact of TET1 on metastasis-associated cellular properties, we examined the sphere-forming abilities of the gastric cancer cells with altered expression of TET1. Non-attached spheres derived from isolated tumor samples or cancer cell lines are believed to derive from cancer stem cells, which are capable of generating complete tumors [4,20,27]. Knocking-down TET1 in NCI-N87 and MKN28 cells increased both the numbers and sizes of spheres derived from single cancer cells (Figure 3A, quantifications in Figure 3B,C). On the contrary, TET1 overexpression strongly decreased the numbers and sizes of spheres of HGC27 and MKN45 cells (Figure 3D, quantifications in Figure 3E,F).

We also examined epithelial-mesenchymal transition (EMT), another essential feature related to metastasizing cancer cells and cancer stem cells. By immunofluorescence staining of cytoskeleton and essential EMT markers, we observed that knocking-down TET1 in MKN28 and AGS cells induced more scattered phenotypes, while overexpressing TET1 promoted epithelial clustering of HGC27 cells G (Figure 3G and Figure S4A). Knocking-down TET1 also strongly reduced the expression of the epithelial cell adhesion molecule E-cadherin, but induced expression of the mesenchymal cytoskeleton protein vimentin and the EMT-promoting transcription factors Snail, Slug, Twist1 and Twist2 (Figure 3G,H,I and Figure S4A). On the contrary, TET1 overexpression enhanced expression of E-cadherin, but reduced expression of mesenchymal markers and EMT-inducing transcription factors (Figure 3H,I). Since EMT is often associated with enhanced cell motility [23,28], we performed transwell migration and invasion assays using cell lines with modulated expression of TET1; we found that knocking-down TET1 promoted transwell migration, while overexpressing TET1 inhibited migration (Figure 3J and Figure S4B). Only MKN28, HGC27 and MKN45 cells were able to invade through Matrigel-coated filters; and MKN28_shTET1 cells showed enhanced invasion, while TET1-overexpressing HGC27 and MKN45 cells exhibited reduced invasion (Figure 3J). These findings demonstrate that knocking-down TET1 in gastric cancer cells produces cell morphologies that are characteristic of cancer stem cells, induce EMT, and enhance cell migration and invasion in culture; overexpression of TET1 inhibits CSCs’ expansion and preserves epithelial properties of gastric cancer cells, and keeps cell motility low.

### 3.4. Inhibitory Effects of TET1 on CSCs and EMT Can Be Reversed by Activating Wnt Signaling

**Figure 4:**
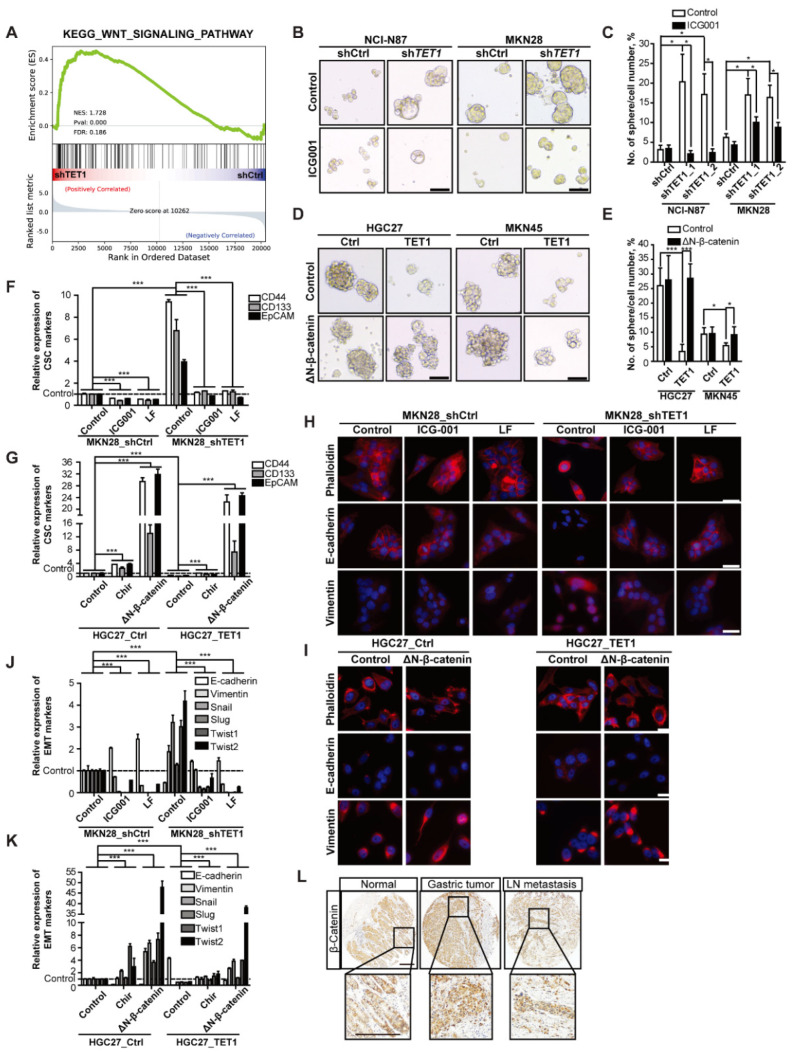
Inhibitory effects of TET1 on CSCs and EMT can be reversed by activating Wnt signaling. (**A**) GSEA analysis of RNA-seq data showing activation of WNT signaling pathway in AGS_shTET1 compared to shCtrl cells. (**B**) Representative pictures of spheres generated by stable NCI-N87 and MKN28 cell lines treated with 25 μmol/L ICG001 or DMSO solvent as control. Scale bars represent 100 µm. (**C**) Numbers of spheres per 100 cells treated as in (**B**). (**D**) Representative pictures of spheres generated by stable HGC27 and MKN45 cell lines transfected with constitutively active ΔN-β-catenin or empty vector as control. Scale bars represent 100 µm. (**E**) Numbers of spheres generated per 100 cells treated as in (**D**). (**F**) qRT-PCR analysis of the cancer stem cell markers in MKN28 stable cell lines treated with 25 μmol/L ICG001, 30 μmol/L LF or DMSO solvent as control. MKN28_shCtrl cells treated with DMSO solvent were used as control. (**G**) qRT-PCR analysis of the cancer stem cell markers in HGC27 stable cell lines treated with DMSO solvent as control, Chir, or transfected with ΔN-β-catenin. HGC27_Ctrl cells treated with DMSO solvent were used as control. (**H**) IF staining of β-actin (by phalloidin), E-cadherin, and vimentin in MKN28 stable cell lines with the indicated treatments. Scale bars represent 100 µm. (**I**) IF staining of β-actin (by phalloidin), E-cadherin, and vimentin in HGC27 stable cell lines with transient transfection. Scale bars represent 100 µm. (**J**) qRT-PCR analysis of the EMT markers in MKN28 stable cell lines as in (**F**). (**K**) qRT-PCR analysis of the EMT markers in HGC27 stable cell lines as in (**G**). (**L**) Representative pictures of β-catenin IHC staining in normal tissue, primary tumors and lymph node metastases of gastric cancer patients. Scale bars represent 100 µm. Error bars indicate mean ± SD. ***, *p* < 0.001. *, *p* < 0.05.

To unveil the molecular mechanisms of how TET1 may inhibit the expansion of cancer stem cells and EMT, we performed RNA-seq analysis of shTET1 cells (see declarations for dataset IDs). The GSEA analysis showed that altered gene expression in shTET1 cells enriched the Wnt signaling pathway (Figure 4A and Figure S5A). We further confirmed the effects of altered TET1 expression on Wnt signaling activity by TOP/FOP flash assays, which showed that knocking-down TET1 increased Wnt signaling (Figure S5B, white bars). The enhanced Wnt signaling could be reversed, although not completely by Wnt inhibitor ICG001 (Figure S5B, black bars). Conversely, TET1 overexpression decreased Wnt signaling, which was rescued by dominant active ΔN-β-catenin (Figure S5C). Remarkably, the numbers and sizes of spheres were upregulated in shTET1-transduced cell lines, while treatment with ICG001 largely reduced the sphere-forming abilities of these cells (Figure 4B, quantified in Figure 4C and Figure S5D). On the contrary, ectopic expression of ΔN-β-catenin could fully rescue the phenotype of TET1 overexpression (Figure 4D, quantified in Figure 4E and Figure S5D).

We further analyzed the CSC markers EpCAM and CD44 by flow cytometry. Knocking-down TET1 increased the frequency of CD44^+^EpCAM^+^ cells, which could be suppressed by the Wnt inhibitors ICG001 or LF [22] (Figure S5E). TET1 overexpression in HGC27 cells decreased the percentage of CSCs significantly, while treatment with the Wnt activator CHIR98014 (hereafter referred to as Chir) [29] or expression of ΔN-β-catenin increased the frequency of CD44^+^EpCAM^+^ cells (Figure S5F). The qRT-PCR analysis of CSC markers confirmed the flow cytometry results (Figure 4F,G).

We also performed immunofluorescence to examine whether modulation of Wnt signaling would impact EMT in TET1-knocking-down or -overexpressing cells. Wnt inhibitors ICG001 or LF rescued the expression of E-cadherin, and reduced the expression of vimentin (Figure 4H and Figure S6A). In contrast, ΔN-β-catenin expression sufficiently rescued the epithelial clustering and vimentin downregulation (Figure 4I). We further performed qRT-PCRs on prominent EMT markers E-cadherin and vimentin and EMT transcription factors Snail, Slug, Twist1, Twist2, Zeb-1, Zeb-2, ID2, together with the classical Wnt target gene Axin2; these assays confirmed the EMT changes and the corresponding epithelial-mesenchymal transition were rescued by the modulation of the Wnt signaling activity (Figure 4J,K and Figure S6B,C).

To further confirm the high activity of Wnt/β-catenin signaling in gastric cancer, we performed β-catenin immunohistochemistry (IHC) in the same tissue arrays as in Figure 1 and analyzed the TCGA dataset. We observed elevated expression of β-catenin in tumor samples and lymph node metastasis (Figure 4L and Figure S4D). The Kaplan–Meier analysis of TCGA gastric cancer patient dataset revealed that high expression of β-catenin was associated with poor overall survival (Figure S6E). Surprisingly, some metastases in distant organs harbored no expression of β-catenin, in contrast to the high levels of β-catenin in the primary tumors (Figure S6F), suggesting that activated Wnt/β-catenin signaling might not be necessary for secondary metastases to grow in proper microenvironments. Taken together, our results showed that Wnt/β-catenin signaling is essential for self-renewal of CSCs and EMT in gastric cancer, and TET1 decreased the canonical Wnt/β-catenin signaling activity to inhibit the metastasis-related cellular properties.

### 3.5. TET1 Inhibits Wnt Signaling through FOXO4 Transactivation

**Figure 5:**
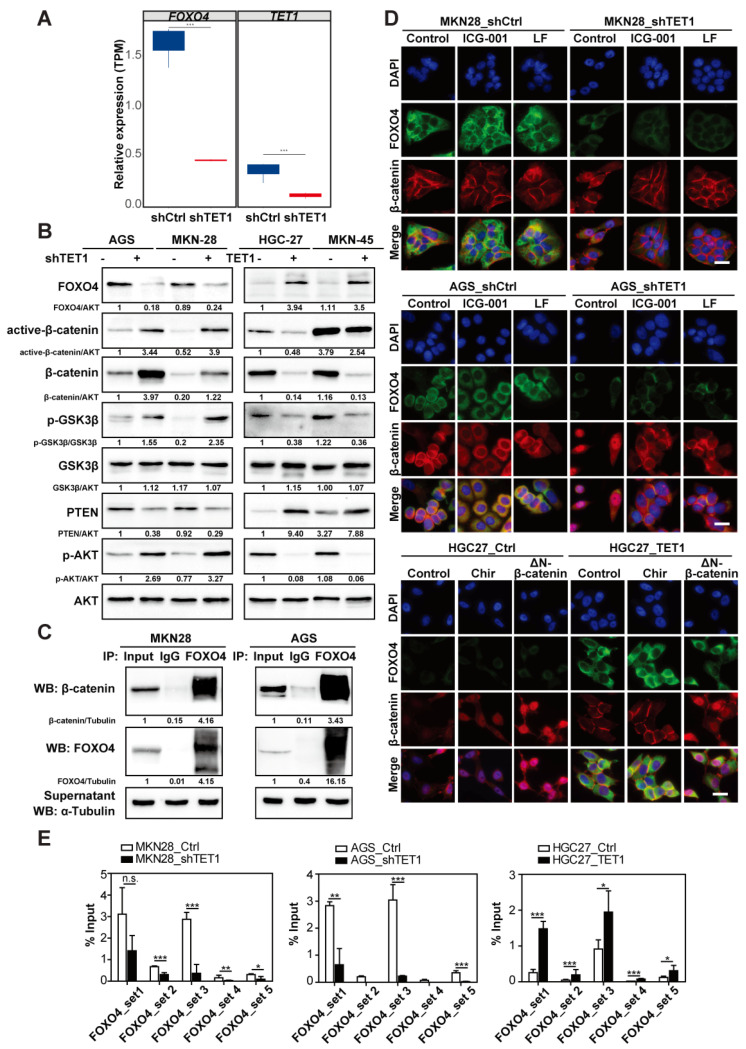
TET1 inhibits Wnt signaling through FOXO4 transactivation. (**A**) RNA-seq analysis of AGS stable cell lines (shTET1 vs. shCtrl) showing differentially expressed TET1 and FOXO4 transcripts. (**B**) Immunoblot analysis of indicated proteins in stable gastric cancer cell lines. (**C**) Immunoprecipitation of MKN28 and AGS cell lysates using anti-FOXO4 antibody, followed by immunoblot analysis of β-catenin and FOXO4. αTubulin was used as loading control. (**D**) IF staining of FOXO4 and β-catenin of MKN28, AGS and HGC27 stable cell lines with the indicated treatments or transient transfection. Scale bars represent 100 µm. (**E**) ChIP-qPCR analysis using an anti-TET1 antibody and PCR primers specific for FOXO4 promoter in MKN28, AGS, and HGC27 stable cell lines. Error bars indicate mean ± SD. ***, *p* < 0.001. **, *p* < 0.01. *, *p* < 0.05. n.s., not significant.

To unveil the molecular mechanisms of how TET1 may inhibit Wnt signaling, we focused on the top changed gene FOXO4 from the RNAseq analysis of shTET1 cells (Figure 5A), due to its known association with β-catenin [30]. Remarkably, FOXO4 expression was low in shTET1 cells and high in TET1-overexpressing cells, and was directly opposite to the expression of active-β-catenin, phosphorylated GSK3β and phosphorylated AKT levels (Figure S7A and Figure 5B,D), suggesting that FOXO4 might work as an upstream suppressor of Wnt and PI3K signaling. By co-immunoprecipitation (co-IP) assay with an anti-FOXO4 antibody, we could observe a direct interaction between FOXO4 and β-catenin in gastric cancer cells (Figure 5C). Immunofluorescence staining also confirmed co-location of cytoplasmic FOXO4 and β-catenin (Figure 5D, see MKN28_ and AGS_shCtrl cells, and HGC27_TET1 cells). Since both ICG-001 and LF are inhibitors targeting the transcription complex of β-catenin, they had little effects on the localization of β-catenin in the MKN28 and AGS control cells; however, upon TET1-knocking-down in AGS cells, which might release β-catenin from FOXO4, treatment with ICG-001 or LF prevented β-catenin nuclear translocation (Figure 5D). Meanwhile, overexpression of ΔN-β-catenin clearly enhanced nuclear β-catenin, even in TET1-overexpressing HGC27 cells (Figure 5D). Taken together, our results suggest that FOXO4 might inhibit Wnt/β-catenin signaling activity by binding to cytoplasmic β-catenin, thus preventing its nuclear translocation.

Next, we asked whether FOXO4 expression was regulated by TET1-induced DNA demethylation. Indeed, the chromatin immunoprecipitation (ChIP) assay showed that TET1 directly bound to the FOXO4 promoter (Figure 5E). Accordingly, TET1 antibodies enriched less chromatin in shTET1 cells and more DNA fragments in TET1-overexpressing cells (Figure 5E). We had previously shown that in SGC7901 gastric cancer cells, TET1 binds to the PTEN promoter and enhances its transcription, and subsequently suppresses PI3K signaling and cell proliferation [31]. We confirmed our finding that TET1 could bind to the PTEN promoter, which also responded to the altered levels of TET1 expression in MKN28 and AGS cells (Figure S7B). Taken together, in line with the GSEA and immunoblot analyses (Figure 4A and Figure 5B), TET1 negatively regulates Wnt/β-catenin and PI3K signaling through demethylating promoters of FOXO4 and PTEN, respectively.

**Figure 6:**
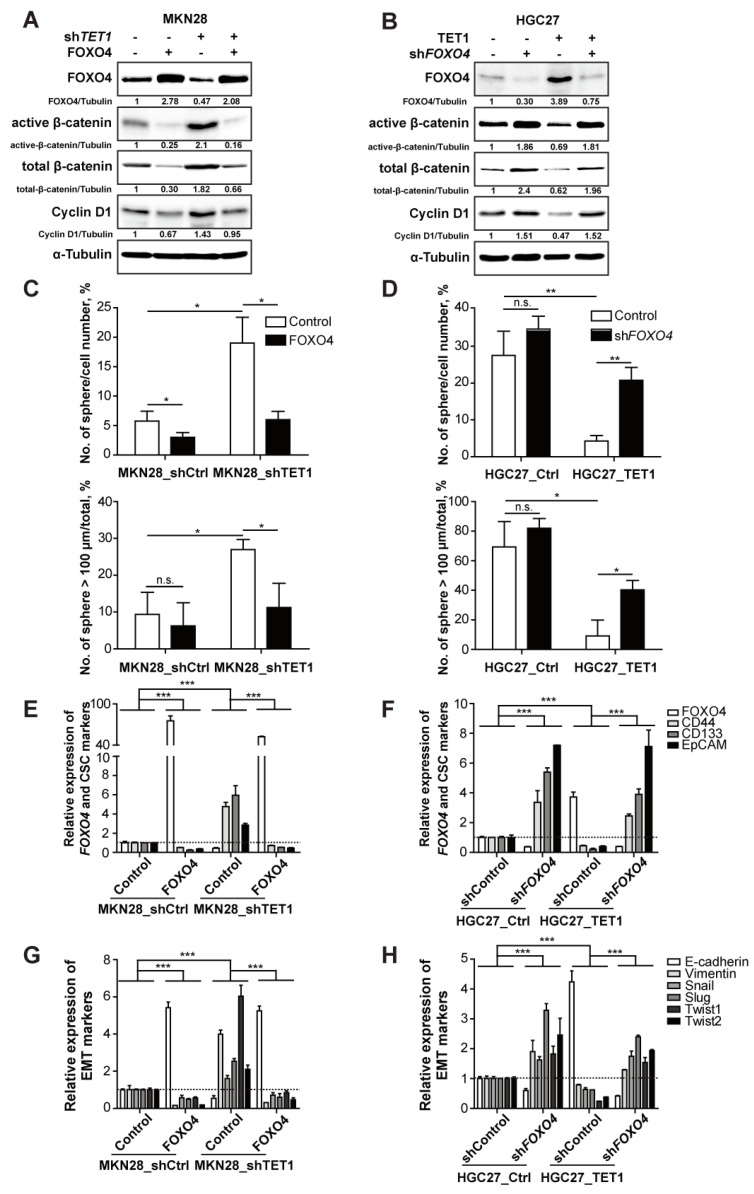
TET1 inhibits Wnt signaling through FOXO4 transactivation. (**A**) Immunoblot analysis of indicated proteins in MKN28 stable cell lines. (**B**) Immunoblot analysis of indicated proteins in HGC27 stable cell lines. (**C**,**D**) Numbers of spheres per 100 cells (**upper**) and percentage of spheres with diameter >100 µm (**lower**) of indicated MKN28 cells (**C**) and HGC27 cells (**D**). (**E**,**F**) qRT-PCR analysis of FOXO4 and cancer stem cell markers in MKN28 cells as in (**A**,**E**) and in HGC27 cells as in (**B**,**F**). (**G**,**H**) qRT-PCR analysis of the EMT markers in MKN28 cells as in (**A**,**G**) and in HGC27 cells as in (**B**,**H**). Error bars indicate mean ± SD. ***, *p* < 0.001. **, *p* < 0.01. *, *p* < 0.05. n.s., not significant.

To further confirm the functions of FOXO4, a downstream demethylating target of TET1, we overexpressed FOXO4 in MKN28_shTET1 cells and knocked-down FOXO4 in HGC27_TET1 cells (Figure 6A,B,E,F, see FOXO4). FOXO4 overexpression reduced active β-catenin in both control and TET1-knocking-down cells (Figure 6A), while knocking-down FOXO4 enhanced levels of active β-catenin in both control and TET1-overexpressing cells (Figure 6B), confirming that FOXO4 is the downstream effector of TET1 that negatively regulates Wnt signaling. Correspondingly, the classical Wnt targets Cyclin D1 and Axin2 showed enhanced expression in TET1-knocking-down cells and reduced expression in TET1-overexpressing cells, both of which were rescued by modulating FOXO4 (Figure 6A,B and Figure S8A,B). Moreover, sphere-culture experiments showed that overexpression of FOXO4 reduced the frequencies of both spheres and large spheres generated by TET1-knocking-down cells (Figure 6C). On the contrary, knocking-down FOXO4 increased sphere numbers and frequencies of large spheres generated by TET1-overexpressing cells (Figure 6D). The rescue effects of knocking-down or overexpressing FOXO4 in TET1-modulated cells also showed in the expression of CSC and EMT markers (Figure 6E–H and Figure S8A,B), which further confirmed that TET1 suppresses cancer metastasis through decreasing the features of CSC and EMT by inhibiting Wnt signaling through transactivating FOXO4.

### 3.6. The Negative Wnt Regulator FOXO4 and the Wnt Target EpCAM Exhibit Prognostic Values

**Figure 7:**
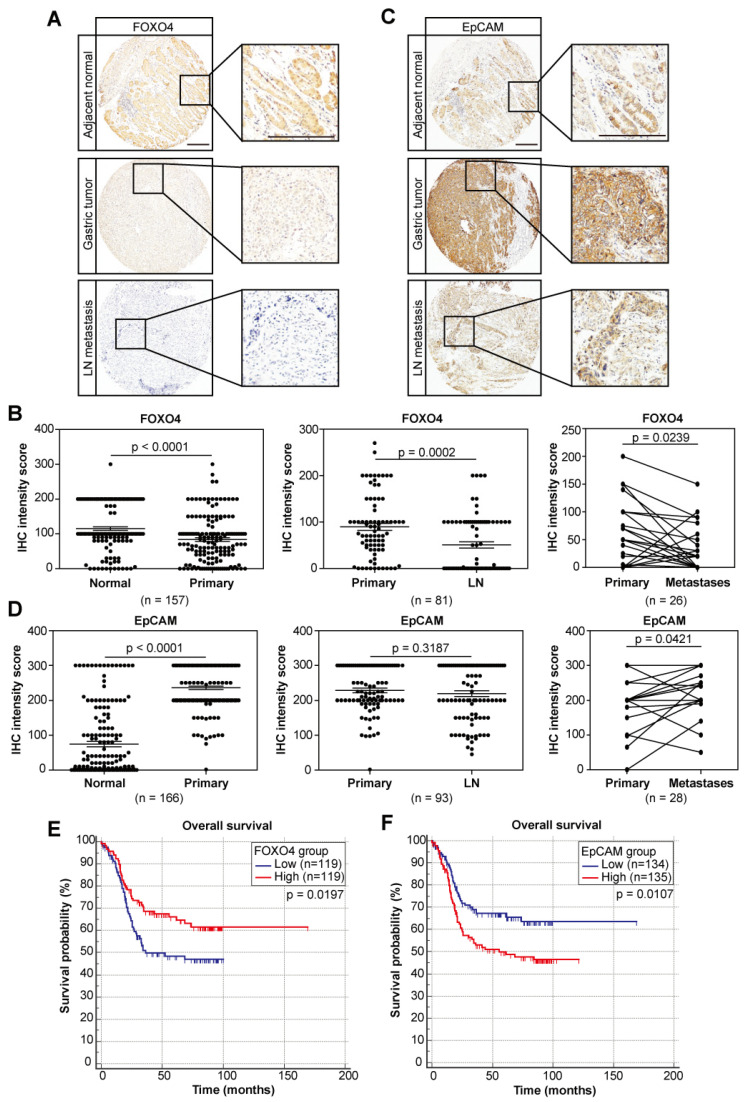
The negative Wnt regulator FOXO4 and the Wnt/β-catenin target gene EpCAM exhibit prognostic values. (**A**,**C**) Representative pictures of FOXO4 (**A**) and EpCAM (**C**) IHC staining in normal tissue, primary tumors and lymph node metastases of gastric cancer patients. Scale bars represent 100 µm. (**B**,**D**) IHC staining intensities comparing FOXO4 (**B**) and EpCAM (**D**) expression in normal tissue samples vs. paired primary tumors (**left**), primary tumors vs. paired lymph node metastases (**middle**), primary tumors vs. paired distant metastases (**right**). Error bars indicate mean ± SEM. (**E**,**F**) Kaplan–Meier analysis showing overall survival of gastric cancer patients with high vs. low FOXO4 (**E**) and EpCAM (**F**) expression. Median value of FOXO4 or EpCAM IHC intensities is used as cut-off to separate low and high groups.

Next, we analyzed the expression of FOXO4, as well as the Wnt/β-catenin target and gastric cancer stem cell marker EpCAM in the human gastric cancer tissue arrays and the TCGA dataset. FOXO4 showed lower expression in the cancerous tissues and even lower expression in lymph node and distant organ metastases (Figure 7A,B and Figure S9A). Kaplan–Meier survival analysis showed that low levels of FOXO4 predicted poor survival of patients (Figure 7E). On the contrary, higher expression of EpCAM was observed in tumors and further increased in distant organ metastases (Figure 7C,D and Figure S9B), and was associated with poor overall survival (Figure 7F). We further confirmed the reduced transcription of FOXO4 and enhanced expression of EpCAM in the tumors of the 16 patients with gastric cancer metastasis in Figure 1 (Figure S9C,D). The TCGA dataset also showed the same expression patterns (Figure S9E,F). Kaplan–Meier analysis confirmed that low levels of FOXO4 predicted poor survival of patients (Figure S9F). Although EpCAM levels showed no prognostic values in the TCGA dataset, analysis confined to five years (60 months) showed correlation with overall survival of patients (Figure S9H). Since our previous results showed that FOXO4 is a demethylating target of TET1, which negatively regulates the Wnt pathway; therefore, correlation analysis of IHC staining scores could confirm the causal relationships between these three genes, showing that FOXO4 expression positively correlated with TET1, while EpCAM expression negatively correlated with FOXO4 (Figure S9I). We also performed univariate and multivariate analysis of all the prognostics factors, including expression of TET1, FOXO4 and EpCAM, using the IHC data and clinical information from the tissue array. To our surprise, stringent analysis showed that only tumor stage (TNM), metastasis status and expression of FOXO4 was independent prognostic factors of patient survival, suggesting that expression of TET1 and EpCAM were associated with any of the three independent factors to predict prognosis (Table S1), and FOXO4 is worthy of further translational exploration as an independent prognostic factor.

## 4. Discussion

### 4.1. TET1 Acts as a Tumor Suppressor in Various Cancers

TET1 catalyzes DNA cytosine demethylation by promoting 5mC conversion to 5hmC, then 5fC and 5caC by TET2 and TET3, respectively [9,32]. Recent research has shown that TET1 can inhibit tumorigenesis and cancer progression. Tet1-deficient mice and Tet1/Tet2 double knockout mice develop B cell lymphoma [33]. Decreased levels of 5hmC and TET1 expression have also been observed in hepatocellular carcinoma and melanoma, in which 5hmC level is associated with tumor progression and overall survival of patients [14,34]. In prostate and breast cancers, TET1 transactivates TIMP2 and TIMP3 to inhibit cancer invasion [15]. In our study, TET1 suppresses cancer metastasis through decreasing the features of CSC and EMT by inhibiting Wnt/β-catenin signaling through transactivating FOXO4. Although the mechanism is different, it is consistent with the previous study in colorectal cancer cells; TET1 also inhibits the Wnt pathway indirectly by promoting the expression of Wnt suppressors DKK3 and DKK4 to, therefore, prohibit cell proliferation [35]. Of note, knockdown of TET1 blocked GGT induced the activation of Wnt/β-catenin pathway through an unknown mechanism in gastric cancer cell lines (GES-1, MGC-803 and SGC-7901) [36]. We observed that TET1 can dramatically influence EMT, self-renewal of CSCs and metastasis of gastric cancer cells without dramatically affecting cell proliferation and viability. Our RNA sequencing analysis shows that knocking-down TET1 activates a variety of signaling pathways, of which the Wnt/β-catenin signaling pathway stood out due to its most significant changes and its widely studied roles in cancer initiation and progression. Taken together, TET1 functions mainly as a tumor suppressor and it reactivates downstream signaling pathways in a tissue-specific manner.

### 4.2. TET1 Restrains Wnt/β-Catenin Signaling by Restoring the Expression of Key Tumor Suppressors

Using gastric cancer cells, we observed that FOXO4 can directly bind to β-catenin, preventing its nuclear translocation and inhibiting its transcriptional activity. It has been shown by others in prostate cancer that FOXO3a, another FOXO family member, downregulates β-catenin by transactivating its targeting microRNAs miR-34b/c and inhibits its transcriptional activity by directly binding to β-catenin [37], suggesting that FOXO protein family members may share broad β-catenin inhibitory functionalities in cancer, although their superiority may differ in different cancers. Cellular oxidative stress simultaneously increases binding between β-catenin and FOXO, leading to enhanced FOXO transcriptional activity and inhibiting TCF transcriptional activity [38]. Our data show that in gastric cancer FOXO4 expression can be induced by TET1, and ChIP-qPCR revealed that FOXO4 is indeed a direct TET1-demethylating target gene. Interestingly, knocking-down FOXO4 in TET1-overexpressing cells or overexpressing FOXO4 in TET1-knocking-down cells fully rescued the features of CSC and EMT in gastric cancer cells, suggesting that FOXO4 might be the major player downstream of TET1 to regulate metastasis-promoting Wnt signaling. A low FOXO4 expression level is also significantly associated with poor patient survival. Stringent multivariate analysis also confirmed that FOXO4, instead of TET1, is an independent prognostic factor predicting patients’ survival, suggesting that the direct regulation of Wnt signaling by FOXO4 might regulate gastric cancer progression in a more profound way than TET1.

Intriguingly, constantly active ΔN-β-catenin is sufficient to rescue the EMT and self-renewal of CSCs in TET1-overexpressing cells, while the GSK3β inhibitor Chir has a much weaker effect. First, it shows that TET1-induced FOXO4 cannot inhibit ΔN-β-catenin activity, which hints that the structural change in β-catenin protein may lead to the failure of FOXO4 binding. In addition, some gastric cancer cell lines used in this study suffer mutations in APC (HGC27, MKN45, and MKN28) or CTNNB1 (AGS). Immunoprecipitation assays showed that FOXO4 could not only interact with wild-type β-catenin in MKN28 cells, but also precipitates with β-catenin mutants (G34E) in AGS cells. Second, the protein interaction affinity of FOXO4 and β-catenin is independent of β-catenin protein activity, and thereby cannot be blocked by Chir-mediated activation of β-catenin. This can partially explain why GSK3β can be indirectly activated by TET1, as GSK3β is directly phosphorylated by AKT and degraded, while AKT is inhibited by TET1 target PTEN [39]. However, we cannot exclude other possible mechanisms, since TET1 regulates a much broader spectrum of gene expression profile, which needs further investigation.

### 4.3. Regulation of TET1 Expression and Enzyme Activity

TET proteins all harbor the same catalytic activity, yet they take part in diverse biological processes, partly due to their differential expression in a tissue-specific manner [9,40]. For example, TET3 is the predominant form expressed in neurons [41], and TET2 is expressed hematopoietic stem cells [42]. In our study, only TET1 showed reduced expression in gastric cancer, while TET2 and TET3 increased in cancer tissues, an expression pattern consistent with the TCGA gastric cancer dataset. The non-catalytic domains of the TET enzymes have been shown to interact with a large number of DNA-binding factors, some of which may recruit them to specific genomic loci [43]. The TET1 CXXC domain has little specificity for CpGs [44], suggesting that other proteins facilitate its localization to CpGs [45]. TET2 completely lacks a CXXC domain and requires IDAX for CpG binding [46]. The CXXC domain of TET3 binds most strongly to 5caC-modified CpGs, and it is found to be enriched on TSSs of a specific set of genes in the neuronal population [47]. TET1 binds the promoter at a region with H3K27me2/3 modification [48]. Therefore, the distinct recruitment mechanisms of TET enzymes to CpGs would play a role in determining their function.

Knowing the essential roles of TET1 in gastric cancer to inhibit metastasis, EMT and self-renewal of CSCs, it is conceivable that reactivating TET1 may be beneficial for gastric cancer patients in the clinic. α-Ketoglutarate (α-KG) is one of the cofactors for TET1 and other demethylases. It has been reported that an injection of a glucose source could promote levels of α-KG, accompanied by increased levels of 5hmC and 5fC [49]. Isocitrate dehydrogenase 1 (IDH1) and isocitrate dehydrogenase 2 (IDH2) catalyze the conversions of isocitrate to α-KG. IDH protein mutants that are commonly discovered in gliomas and AML [50,51,52,53] are able to convert α-KG to 2HG and inhibit α-KG-dependent enzyme activity, such as TET1. In myeloid malignancies, IDH mutants, which are believed to be responsible for decreased levels of global 5hmC and poor patient survival [54], may serve as novel targets for further exploration.

In gastric cancer, however, IDH mutants are rather rare, implying that restoration of TET1 expression might be more important than its enzyme activity. It has been reported that miR-22 directly targets and reduces TET1 expression, which results in significantly lower global 5hmC and promotes breast cancer EMT and metastasis in mouse xenograft models [55], making itself a potential target for cancer therapies. In another study, we observed in triple negative breast cancer that TET1 is repressed by polycomb repressive complex 2 (PRC2)-mediated H3K27me3 silencing [56]. Inhibition of PRC2 core factor EZH2 by a specific inhibitor was able to restore TET1 expression and suppress TNBC cell propagation, providing us with a novel strategy for TET1 induction and tumor suppression. Overall, the present data show that TET1 inhibits gastric cancer metastasis, potentially by directly transactivating FOXO4 and confining β-catenin in the cytoplasm, thus reducing EMT and self-renewal of CSCs. So, it will also be interesting to examine whether knocking-down FOXO4 in MKN28 and FOXO4 overexpression in MKN45 will phenocopy the effects of TET1 on metastasis in an in vivo metastasis model.

## 5. Conclusions

In this study, we unveiled a novel TET1-FOXO4-β-catenin signaling cascade, in which TET1 inhibits β-catenin activity and its nuclear translocation through transactivating FOXO4 expression. TET1 expression can significantly inhibit EMT and stemness properties of gastric cancer cells, while knocking-down endogenous TET1 induces metastasis and enhances self-renewal of CSCs by activating canonical Wnt signaling, which could be fully rescued by modulating FOXO4 expression. Our data also showed that low expression of TET1 or FOXO4 predicts poor survival of gastric cancer patients, suggesting that reactivation of TET1 or FOXO4 might be a novel therapeutic approach to prevent gastric cancer metastasis.

## Data Availability

RNA sequencing data have been deposited in NCBI-BioSample database with the following IDs: SAMN10068006, SAMN10068007, SAMN10068008, SAMN10068009, SAMN10068010, and SAMN10068011. Raw data of Western Blot presented in Figure S10. Other raw data presented in this study are available upon request from the corresponding author.

## Supplementary Materials

The following are available online at https://www.mdpi.com/article/10.3390/cancers14133232/s1; Figure S1: related to Figure 1. The DNA methylcytosine dioxygenase TET1 is low in metastatic gastric tumors and predicts poor survival of patients; Figure S2: related to Figure 2. Knocking-down TET1 promotes experimental metastasis, while TET1 overexpression inhibits metastasis in the liver; Figure S3: related to Figure 2. Knocking-down TET1 promotes experimental metastasis, while TET1 overexpression inhibits metastasis in the liver; Figure S4: related to Figure 3. TET1 inhibits self-renewal of cancer stem cells and epithelial-mesenchymal transition; Figure S5: related to Figure 4. Inhibitory effects of TET1 on CSCs and EMT can be reversed by activating Wnt signaling; Figure S6: related to Figure 4. Inhibitory effects of TET1 on CSCs and EMT can be reversed by activating Wnt signaling; Figure S7: related to Figure 5. TET1 inhibits Wnt signaling through FOXO4 transactivation; Figure S8: related to Figure 6. TET1 inhibits Wnt signaling through FOXO4 transactivation; Figure S9: related to Figure 7. The negative Wnt regulator FOXO4 and the Wnt/β-catenin target gene EpCAM exhibit prognostic values; Figure S10: Raw data of Western Blot; Table S1: Univariate and multivariate analyses of prognostic factors for overall survival of gastric cancer patients; Table S2: Antibody list; Table S3: ChIP qRT-PCR primer list.
