# Supplementary material for: Targeting Wnt/β-Catenin Signaling by TET1/FOXO4 Inhibits Metastatic Spreading and Self-Renewal of Cancer Stem Cells in Gastric Cancer
Source: Cancers (Basel). 2022 Jun 30;14(13):3232. doi: 10.3390/cancers14133232 (PMC9264907; doi:10.3390/cancers14133232)
Supplement: Supplementary file 1 [file cancers-14-03232-s001.zip › cancers-1786541-supplementary.pdf]

Qi et al. "Targeting Wnt/β-catenin signaling by TET1/FOXO4 inhibits metastatic spreading and self-renewal of cancer stem cells in gastric cancer"

- Supplementary information -

There are 10 supplementary figures and 3 supplementary tables.

Figure S1, related to Figure 1. The DNA methylcytosine dioxygenase TET1 is low in metastatic gastric tumors and predicts poor survival of patients

(A) RNA-seq analysis of TCGA gastric cancer dataset STAD, showing *TET1*, *TET2* and *TET3* expression in normal tissue vs. tumor samples. (B) Representative pictures of TET1 IHC staining in primary tumors vs. paired distant metastases. Scale bars represent 100 μm.

Figure S2, related to Figure 2. Knocking-down *TET1* promotes experimental metastasis, while TET1 overexpression inhibits metastasis in the liver

(A) qRT-PCR analysis of *TET1* transcripts in indicated gastric cancer cell lines. (B) Immunoblot analysis showing TET1 expression as in *A*. α-Tubulin was used as loading control. (C) Dot blot analysis of global 5hmC and 5mC levels as in *A*. (D) Quantification of 5hmC/5mC levels in *C*. (E, F) qRT-PCR analysis of the *TET1* transcripts in stable cell lines transduced with shCtrl or sh*TET1* (E), or with TET1 or empty vector (Ctrl) (F). (G, I) Dot blot analysis of global 5hmC and 5mC levels in cells as in *E* (G) or as in *F* (I). (H, J) Quantification of 5hmC/5mC levels in *G* (H) and *I* (J).

Figure S3, related to Figure 2. Knocking-down *TET1* promotes experimental metastasis, while TET1 overexpression inhibits metastasis in the liver

(A, B) qRT-PCR analysis of TET1 transcripts in tumor samples from Figure 2E (A) and Figure 2G (B). Spleen tumors generated by MKN28 cells or MKN45 cells were used as control. N.D. represents not detectable. Error bars indicate mean ± SD through this figure.

Figure S4, related to Figure 3. TET1 inhibits self-renewal of cancer stem cells and epithelial-mesenchymal transition

(A) Immunofluorescent staining of Vimentin and β-actin (by Phalloidin) in AGS stable cell lines. Scale bars represent 100 μm. (B) Representative pictures of transwell migration assays with or without Matrigel coating using HGC27 stable cell lines.

Figure S5, related to Figure 4. Inhibitory effects of TET1 on CSCs and EMT can be reversed by activating Wnt signaling

(A) Top scored upregulated GO terms enriched by differentially expressed RNAs (sh*TET1* vs. shCtrl). (B) TOP/FOP-Flash assays of NCI-N87, MKN28, and AGS stable cell lines with the indicated treatments. (C) TOP/FOP-Flash assays of HGC27 and MKN45 stable cell lines with indicated transient transfections. (D) Percentage of spheres with diameter > 100 μm treated as in *Figure 4B* (*left*) or as in *Figure 4D (right)*. (E, F) Flow cytometry analysis showing CD44 and EpCAM expression in MKN28 and NCI-N87 stable cell lines with indicated treatments (E) and in HGC27 stable cell lines with indicated treatments or transient transfection (F). Numbers indicate percentages of double positive cells.

Figure S6, related to Figure 4. Inhibitory effects of TET1 on CSCs and EMT can be reversed by activating Wnt signaling

(A) IF staining of β-actin (by Phalloidin), E-cadherin, and Vimentin in MKN28 and AGS stable cell lines with the indicated treatments. Scale bars represent 100 μm. (B) qRT-PCR analysis of the EMT markers in MKN28 stable cell lines as in *Figure 4F*. (C) qRT-PCR analysis of the EMT markers in HGC27 stable cell lines as in *Figure 4G*. (D) RNA-seq analysis of TCGA gastric cancer dataset STAD, showing β-catenin expression in normal tissue vs. tumor samples. (E) Kaplan-Meier analysis showing overall survival of gastric cancer patients in TCGA-STAD with high vs. low β-catenin expression. (F) Representative pictures of β-catenin IHC staining in primary tumors vs. paired distant metastases. Scale bars represent 100 μm.

Figure S7, related to Figure 5. TET1 inhibits Wnt signaling through *FOXO4* transactivation

(A) qRT-PCR analysis of FOXO4 transcripts in MKN28 and HGC27 stable cell lines. (B) ChIP qPCR analysis using an anti-TET1 antibody and PCR primers specific for *PTEN* promoter in MKN28 and AGS stable cell lines.

Figure S8, related to Figure 6. TET1 inhibits Wnt signaling through *FOXO4* transactivation

(A, B) qRT-PCR analysis of WNT target genes and EMT markers in MKN28 cells as in *Figure 6A* (*A*) and in HGC27 cells as in Figure 6B (*B*). ***, $p < 0.001$.

Figure S9, related to Figure 7. The negative Wnt regulator FOXO4 and the Wnt/β-catenin target gene EpCAM exhibit prognostic values

(A, B) Representative pictures of FOXO4 (*A*) and EpCAM (*B*) IHC staining in primary tumors vs. paired distant metastases. Scale bars represent 100 µm. (C, D) qRT-PCR analysis of *FOXO4* (*C*) and *EPCAM* (*D*) transcripts comparing paired human gastric tumors vs. normal tissue samples as in *Figure 1A*. Error bars indicate mean ± SEM. (E, G) *FOXO4* (*E*) and EpCAM (*G*) expression in normal tissue vs. tumor samples in TCGA-STAD dataset. (F, H) Kaplan-Meier analysis showing overall survival of gastric cancer patients in TCGA-STAD with high vs. low *FOXO4* (*F*) and *EPCAM* (*H*) expression. (I) IHC staining intensities of FOXO4 in patient samples of TET1 low *vs*. high groups (*left*) and EpCAM in patient samples of FOXO4 low *vs*. high groups (*right*). Error bars indicate mean ± SEM through this figure. ***, $p < 0.001$. *, $p < 0.05$.

Figure S10. Raw data of Western Blot.

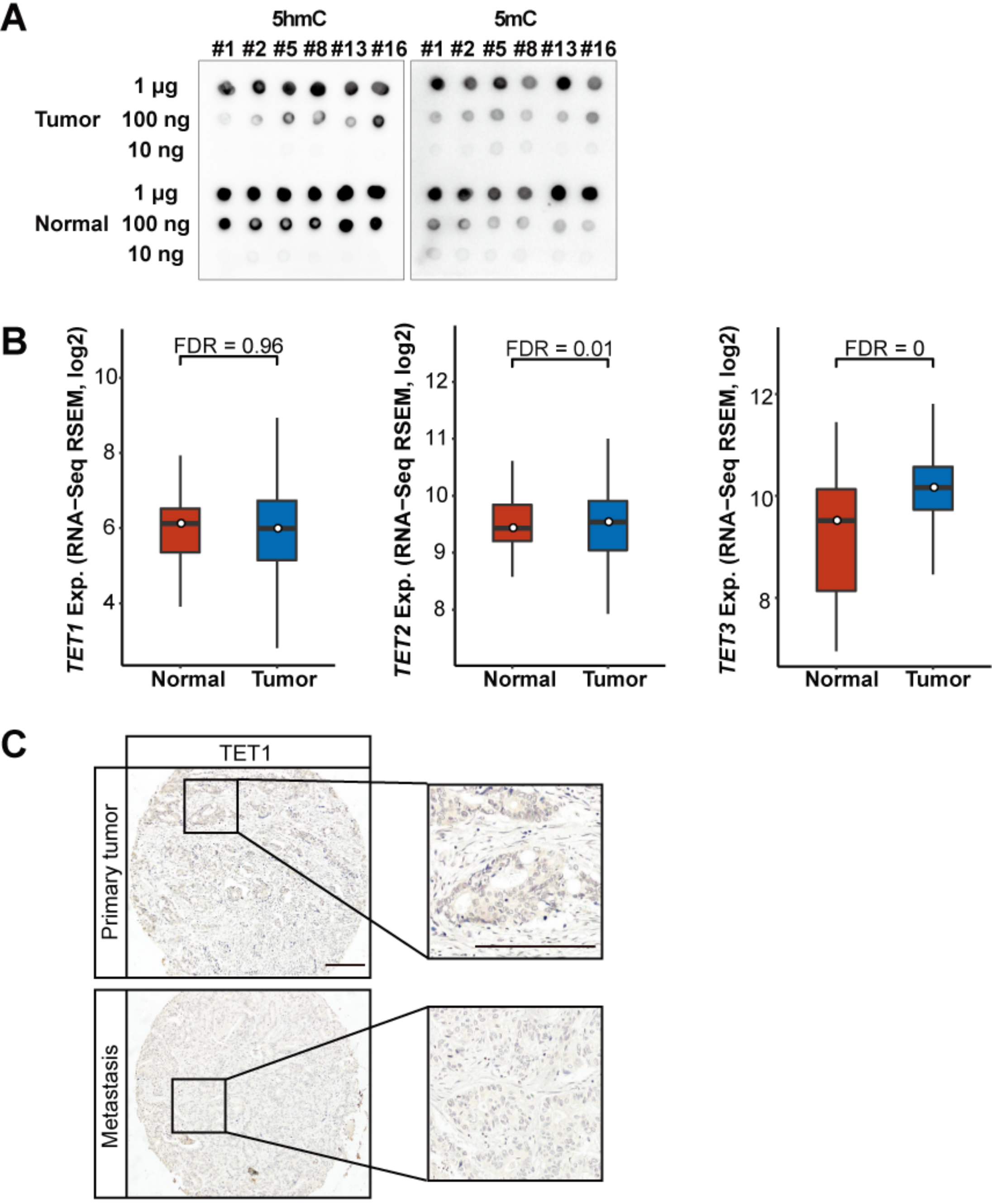

Figure S1. Qi, et al.

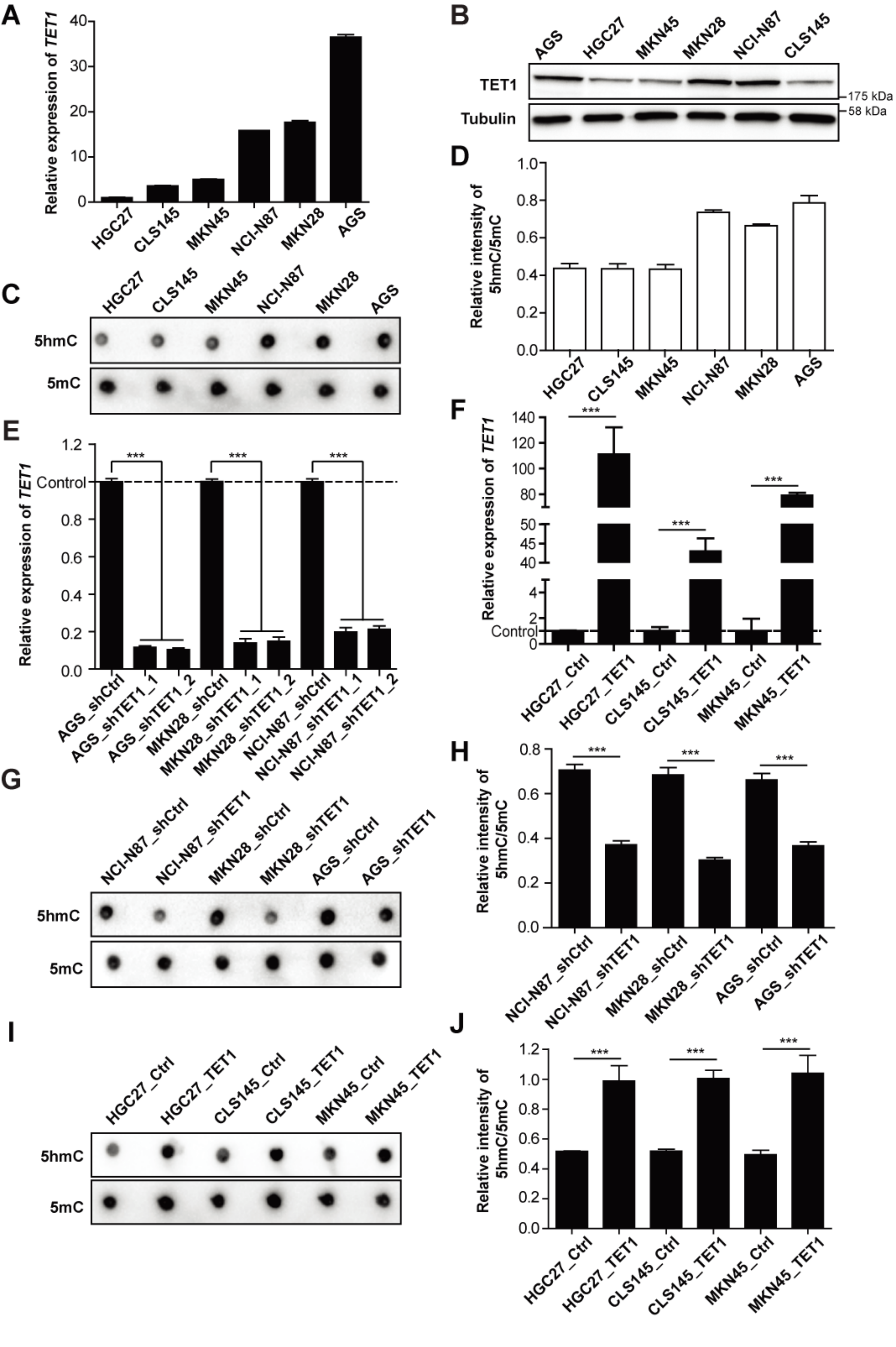

Figure S2. Qi, et al.

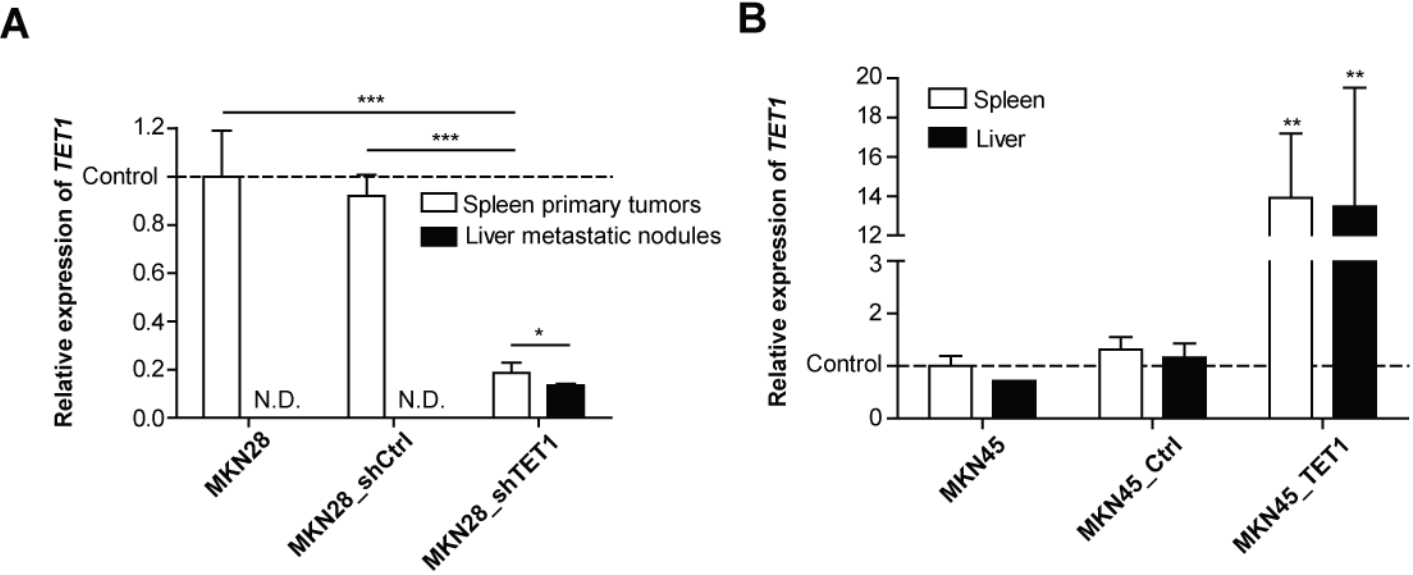

Figure S3. Qi, et al.

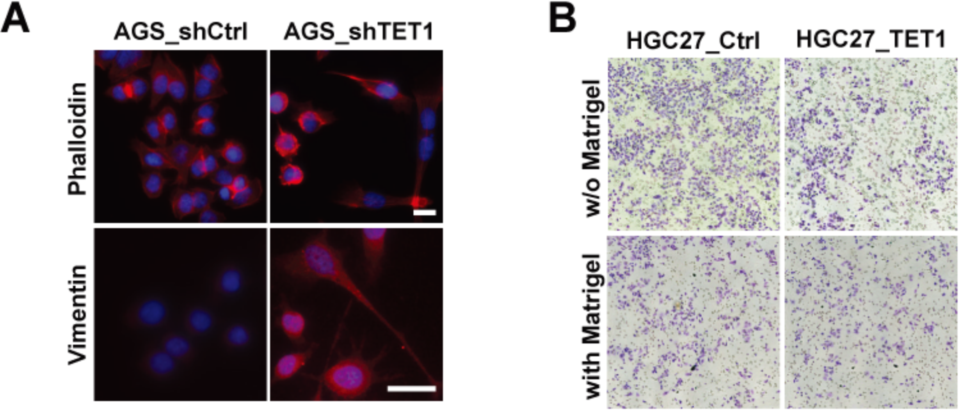

Figure S4. Qi, et al.

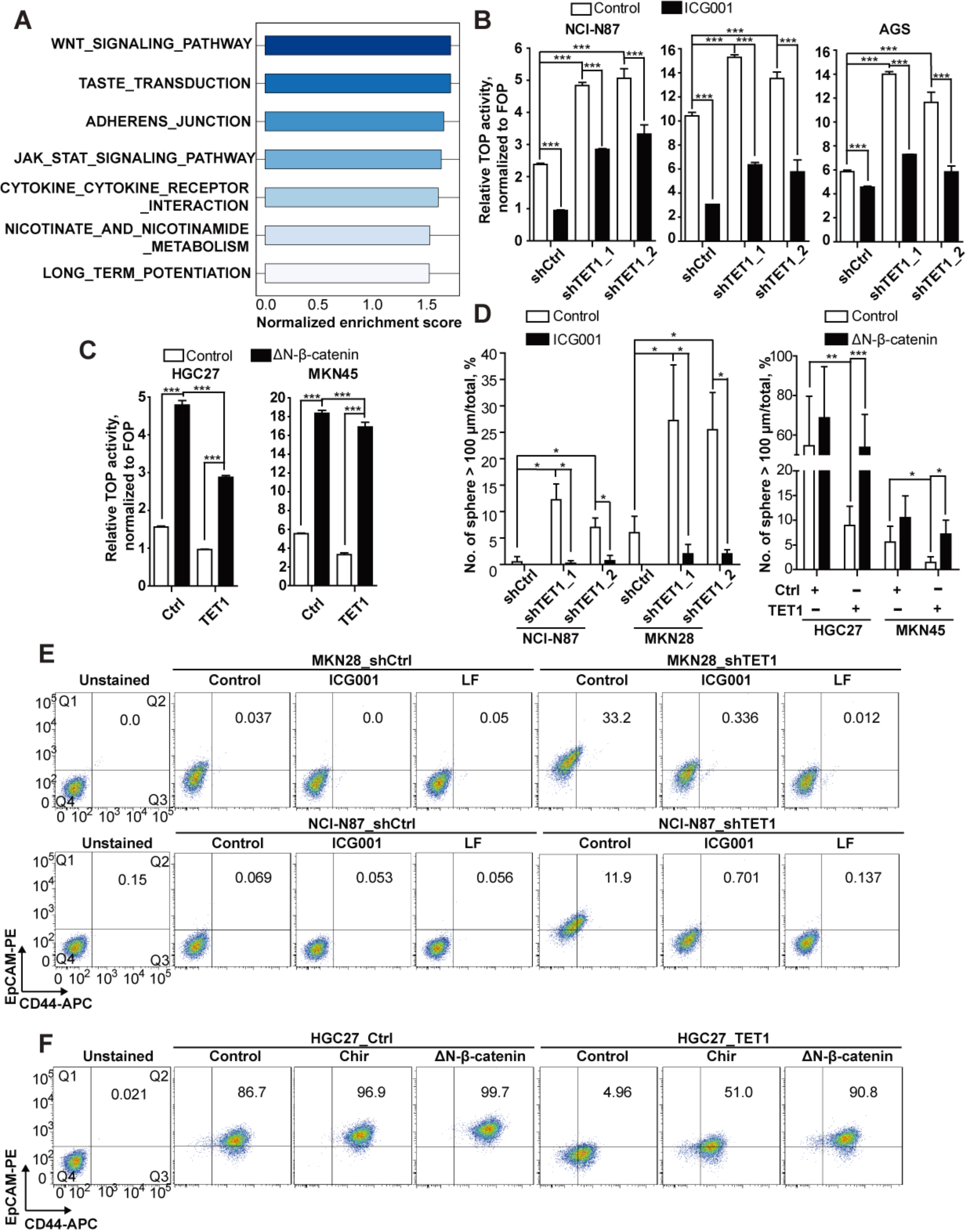

Figure S5. Qi, et al.

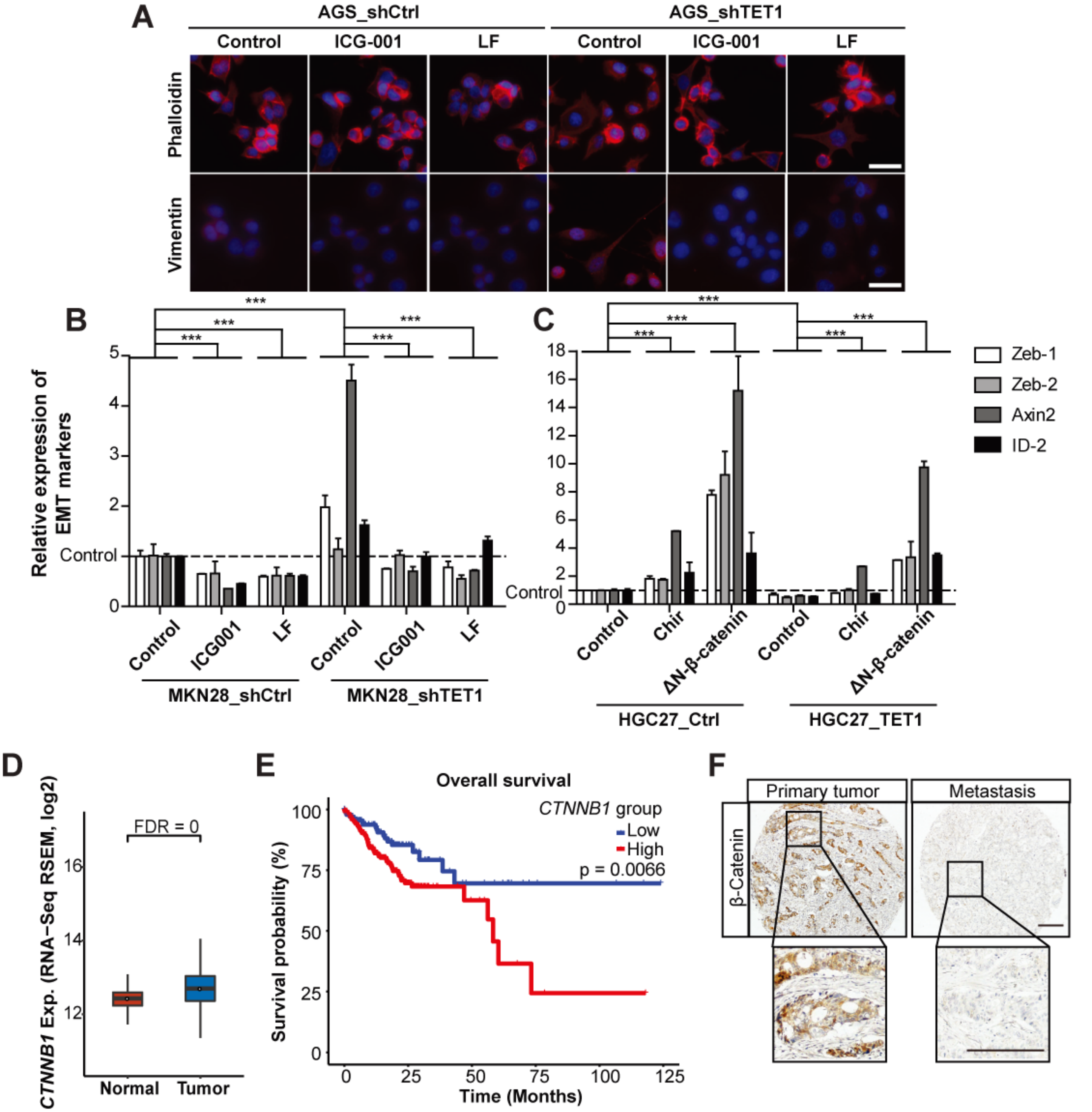

Figure S6. Qi, et al.

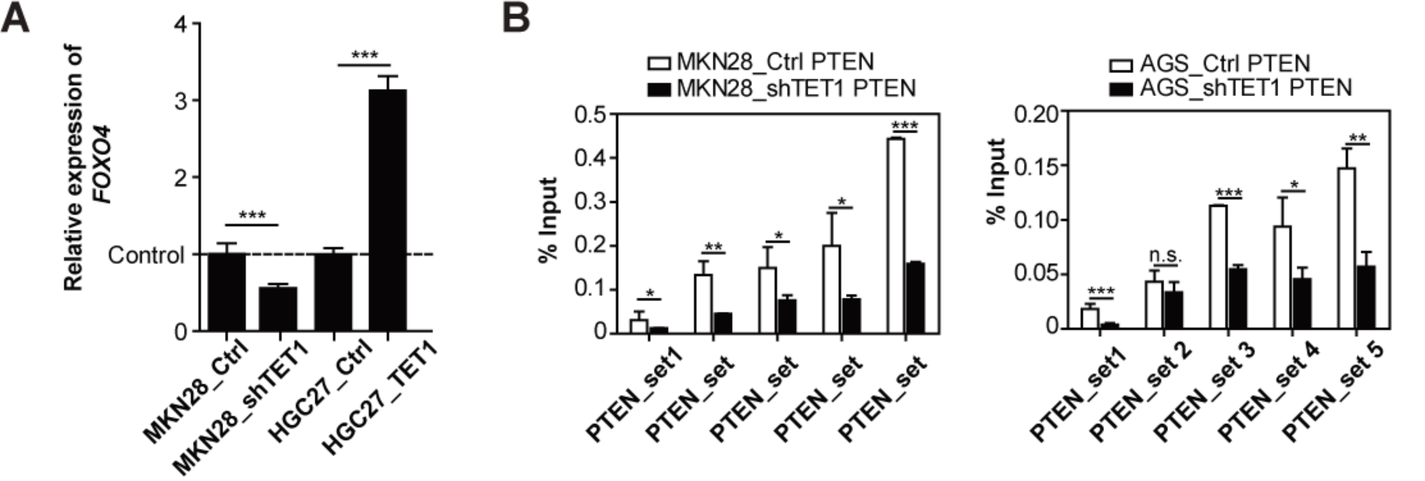

Figure S7. Qi, et al.

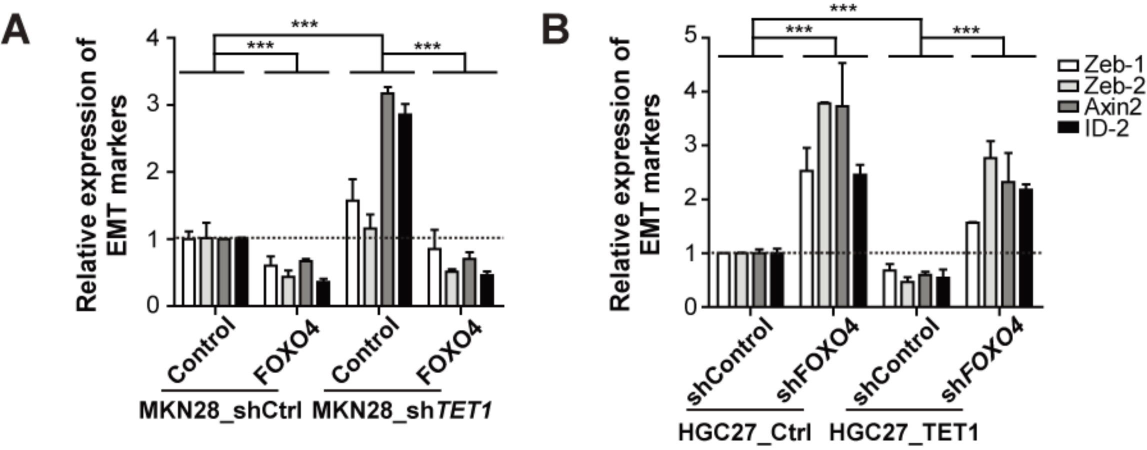

Figure S8. Qi, et al.

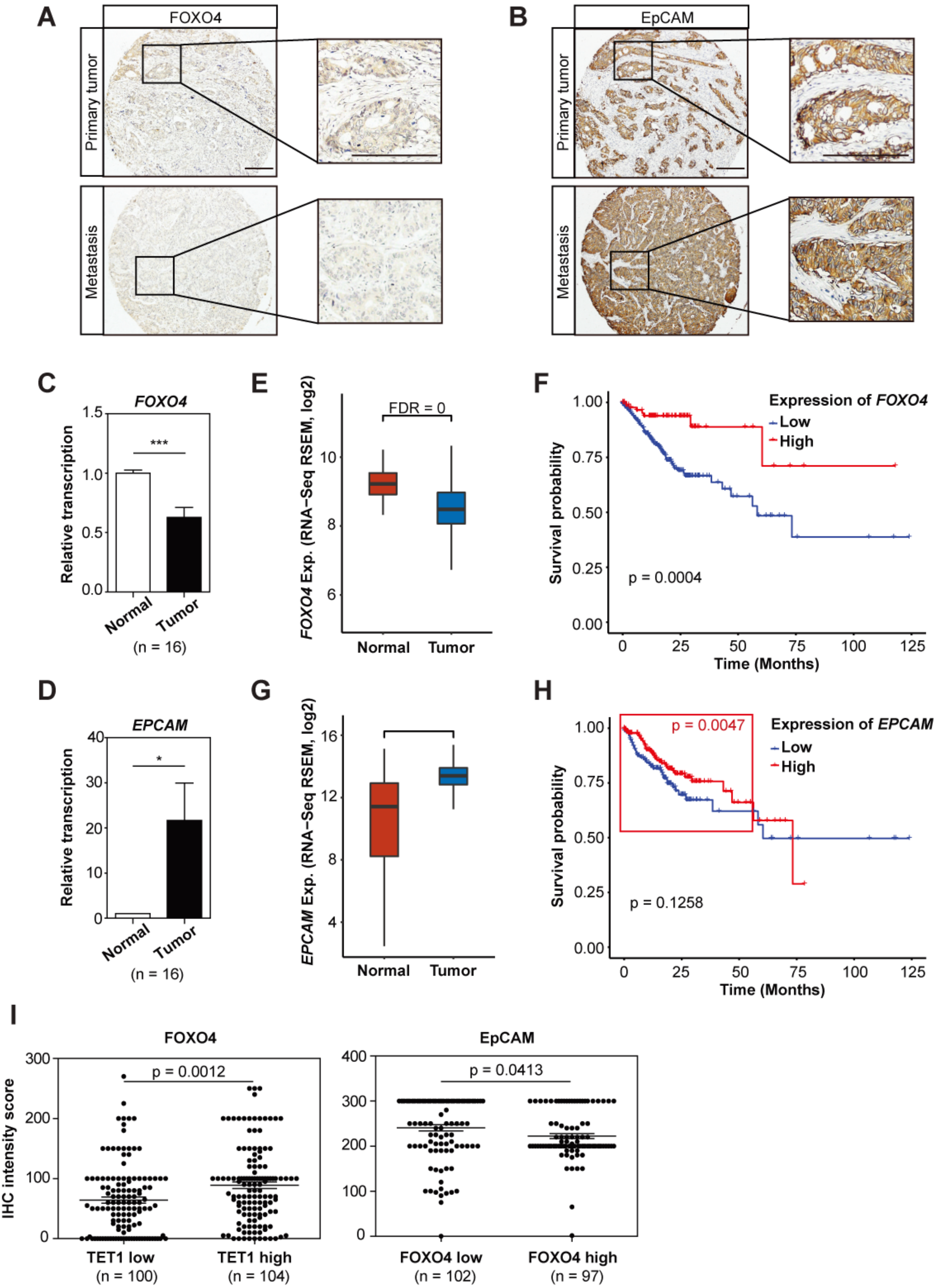

Figure S9. Qi, et al.

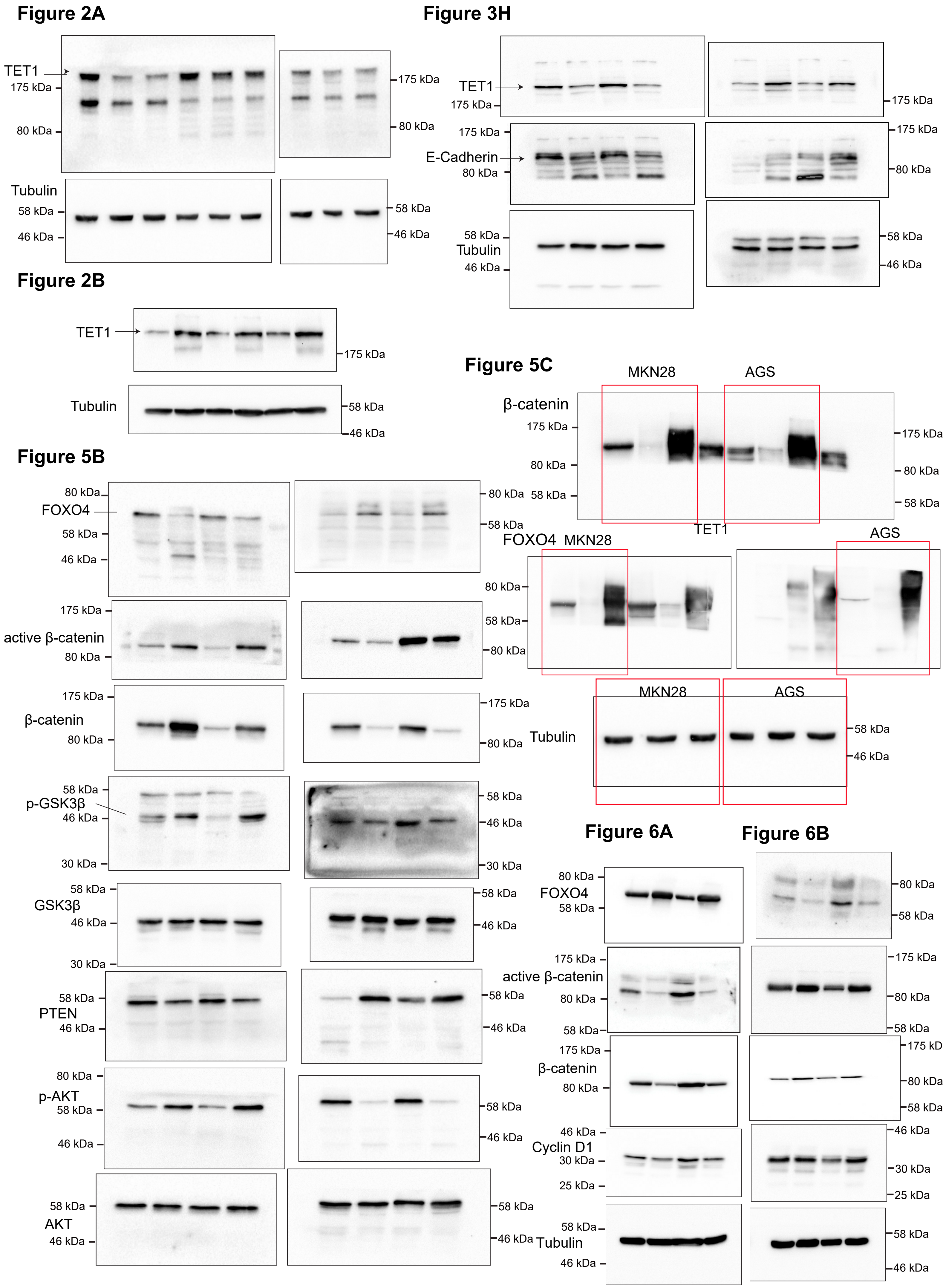

Figure S10. Qi, et al.

Supplementary Table S1: Univariate and multivariate analyses of prognostic factors for overall survival of gastric cancer patients

**Variable**	**Univariate**		**Multivariate**	
	Log-rank		HR	*P* value
	χ^2	P value		
**Gender (male/female)**	2.474	0.116		
**Age, years (> 56/≤ 56)**	0.674	0.412		
**Lymph node metastasis (Yes/No)**	22.393	<0.001	1.371	0.480
**Vascular cancer embolus (Yes/No)**	23.732	<0.001	0.834	0.531
**Pathology differentiation (Well and moderately differentiated/poor differentiation)**	0.738	0.390		
**TNM (I-II/III-IV)**	43.181	<0.001	4.167	<0.001
**Invasion depth (Whole layer/ no whole layer)**	8.132	0.004	0.956	0.925
**Nerve invasion (yes /no)**	13.906	<0.001	0.749	0.450
**Metastasis status (yes/no)**	45.196	<0.001	2.854	<0.001
**TET1 (high expression/low expression)**	4.233	0.040	0.918	0.739
**EpCAM (high expression/low expression)**	6.511	0.011	1.405	0.178
**FOXO4 (high expression/low expression)**	5.443	0.020	0.558	0.021

Supplementary Table S2: Antibody list

Antibody	Application	Manufacturer	Catalog No.
**TET1**	WB 1:1000; ChIP 1:50; IHC 1:200	Novusbiologicals	NBP2-15135
**α-Tubulin**	WB 1:2000	Sigma	T5168
**total H3**	WB 1:2000	Cell signaling technology	4499S
**E-Cadherin**	WB 1:1000; IF 1:200	BD Biosciences	610182
**Cyclin A**	WB 1:1000	Sigma	C4710
**FOXO4**	WB 1:1000; IP 1:50; IF 1:100; IHC 1:500	Cell signaling technology	2499S
**Vimentin**	IF 1:100	Sigma	V6630
**total β-catenin**	WB 1:1000; IF 1:100	Cell signaling technology	8480S
**active-β-catenin**	WB 1:1000	Cell signaling technology	8814S
**EpCAM**	WB 1:1000; IHC 1:200	Cell signaling technology	2929S
**EpCAM-PE**	Flow 1:100	BD Biosciences	347198
**CD44-APC**	Flow 1:100	BD Biosciences	559942
**total AKT**	WB 1:2000	Cell signaling technology	9272S
**p-AKT**	WB 1:1000	Cell signaling technology	4060S

**total GSK**	WB 1:2000	Cell signaling technology	12456S
**p-GSK**	WB 1:1000	Cell signaling technology	5558S
**PTEN**	WB 1:1000	Cell signaling technology	9188S
**IgG**	IP 1:50; ChIP 1:100	Cell signaling technology	2729S
**5hmC**	Dot blot 1:1000	Cell signaling technology	51660S
**5mC**	Dot blot 1:1000	Cell signaling technology	28692S
**HRP-goat-anti-rabbit**	WB 1:1000	GE Healthcare	RPN4301
**HRP-sheep-anti-mouse**	WB 1:2000	GE Healthcare	NA931V

Supplementary Table S3: ChIP qRT-PCR primer list

	Forward/Reverse	Sequence
***FOXO4*-1**	Forward	GTAGCCAGAGGATAGGGGTGC
	Reverse	ACATCTCCAACGGCTTCACTT
***FOXO4*-2**	Forward	GAAAGGGGGAAAGGGCGAAA
	Reverse	CGTGGCAGGATATAACGAGGT
***FOXO4*-3**	Forward	CGGCGAGTGGTAAACAGAGA
	Reverse	CGCACCGATTCCCCTATCAA
***FOXO4*-4**	Forward	GGGGGCAGCAACTTAAAAGGG
	Reverse	TTCTGCAACCTCTCACTCAGC
***FOXO4*-5**	Forward	AGGTCCAACTCCACGTATGG
	Reverse	CTAGGTCTATGATCGCGGCA
***PTEN*-1**	Forward	CCCCGAGCAAAGGAAGAAGA
	Reverse	GGAGGTGGAAGGATACACGG
***PTEN*-2**	Forward	ATCCATCCTGCCGGGTTTTC
	Reverse	CCCCAAGAGAGTCGAGCATC
***PTEN*-3**	Forward	TTTTAGGGCAAACGAGCCGA
	Reverse	GCCAGCGTGTATCACCTCAT
***PTEN*-4**	Forward	CATCATCAGTCCTCCACCCC
	Reverse	CAACGCGAGGCGAGGATAA
***PTEN*-5**	Forward	GCCCGAGCAAGCCCC
	Reverse	GAGCGCAGAGTCCCCAAG
